# Fingerprints of decreased cognitive performance on fractal connectivity dynamics in healthy aging

**DOI:** 10.1007/s11357-023-01022-x

**Published:** 2023-12-20

**Authors:** Zalan Kaposzta, Akos Czoch, Peter Mukli, Orestis Stylianou, Deland Hu Liu, Andras Eke, Frigyes Samuel Racz

**Affiliations:** 1 Department of Physiology, Semmelweis University, 37-47 Tuzolto Street, Budapest, 1094 Hungary; 2 Oklahoma Center for Geroscience and Healthy Brain Aging, University of Oklahoma Health Sciences Center, Oklahoma City, OK USA; 3 Vascular Cognitive Impairment and Neurodegeneration Program, Department of Neurosurgery, University of Oklahoma Health Sciences Center, Oklahoma City, OK USA; 4 International Training Program in Geroscience, Doctoral School of Basic and Translational Medicine/Department of Public Health, Semmelweis University, Budapest, Hungary; 5 Institute of Translational Medicine, Semmelweis University, Budapest, Hungary; 6 Berlin Institute of Health at Charité, University Hospital Berlin, Charitéplatz 1, 10117 Berlin, Germany; 7 Department of Neurology with Experimental Neurology, Charité-University Hospital Berlin, Corporate Member of Freie Universität Berlin and Humboldt Universität zu Berlin, Berlin, Germany; 8 Chandra Department of Electrical and Computer Engineering, Cockrell School of Engineering, The University of Texas at Austin, Austin, TX USA; 9 Department of Radiology and Biomedical Imaging, Yale University School of Medicine, New Haven, CT USA; 10 Department of Neurology, Dell Medical School, The University of Texas at Austin, 1601 Trinity St, Austin, TX 78712 USA; 11 Mulva Clinic for the Neurosciences, Dell Medical School, The University of Texas at Austin, Austin, TX USA

**Keywords:** Fractal connectivity, Detrended cross-correlation analysis, Healthy aging, Cognitive decline, Electroencephalography

## Abstract

Analysis of brain functional connectivity (FC) could provide insight in how and why cognitive functions decline even in healthy aging (HA). Despite FC being established as fluctuating over time even in the resting state (RS), dynamic functional connectivity (DFC) studies involving healthy elderly individuals and assessing how these patterns relate to cognitive performance are yet scarce. In our recent study we showed that fractal temporal scaling of functional connections in RS is not only reduced in HA, but also predicts increased response latency and reduced task solving accuracy. However, in that work we did not address changes in the dynamics of fractal connectivity (FrC) strength itself and its plausible relationship with mental capabilities. Therefore, here we analyzed RS electroencephalography recordings of the same subject cohort as previously, consisting of 24 young and 19 healthy elderly individuals, who also completed 7 different cognitive tasks after data collection. Dynamic fractal connectivity (dFrC) analysis was carried out via sliding-window detrended cross-correlation analysis (DCCA). A machine learning method based on recursive feature elimination was employed to select the subset of connections most discriminative between the two age groups, identifying 56 connections that allowed for classifying participants with an accuracy surpassing 92%. Mean of DCCA was found generally increased, while temporal variability of FrC decreased in the elderly when compared to the young group. Finally, dFrC indices expressed an elaborate pattern of associations—assessed via Spearman correlation—with cognitive performance scores in both groups, linking fractal connectivity strength and variance to increased response latency and reduced accuracy in the elderly population. Our results provide further support for the relevance of FrC dynamics in understanding age-related cognitive decline and might help to identify potential targets for future intervention strategies.

**Supplementary Information:**

The online version contains supplementary material available at 10.1007/s11357-023-01022-x.

## Introduction

Aging has an adverse effect on cognitive functioning even in the absence of identifiable medical pathologies [1, 2]. The workings of this phenomenon are immensely complex and multi-factorial, with many related physiological factors that might contribute to the age-dependent loss of cognitive capabilities [3–6]. Among these, functional connectivity (FC) of the brain has also been established to undergo age-related changes [7], which are often associated with the extent of age-related cognitive decline [8–10]. Even though the origin and impact of these alterations in FC are yet to be fully understood, identifying characteristic connectivity patterns of age-related cognitive impairment is essential for multiple purposes. Biomarkers such as FC could play a pivotal role in the early detection of the onset of cognitive decline, when possible interventions could be more effective. Furthermore, they have the potential to differentiate between the “natural” age-related loss in cognitive functions and impairments caused by pathological conditions such as mild cognitive impairment (MCI) [11] or Alzheimer’s disease (AD) [12]. Nevertheless, to achieve such goals first we must map out how FC changes in relation to age in the healthy population and identify those characteristics that are associated with diminished functionality of various cognitive domains.

Adding to the expanding toolset of FC analyses, fractal connectivity (FrC) has been proposed recently [13]. In essence, instead of estimating the strength of cooperation between distinct brain regions at a single time scale (as with most traditional FC approaches), FrC captures if the strength of connectivity expresses long-term memory in its temporal evolution. Since its introduction, many studies confirmed that FC is not only a dynamic phenomenon [14], but in fact its fluctuations follow a scale-free (or fractal) temporal pattern [15–18], a characteristic that can be affected under various pathological conditions such as AD [19] or schizophrenia [20]. Furthermore, since fractal neural dynamics in general were found associated with both aging [21–23] and cognitive functioning [24, 25], it could be hypothesized that an FrC approach might be viable in linking age-related changes in connectivity to manifestations of cognitive decline. Indeed, it has been recently reported that not only the fractal scaling exponent of functional connections show a general decrease in healthy elderly when compared to young individuals, but many of these distinctive features correlated with reduced cognitive performance in the former group [26].

Although the work presented by Czoch and colleagues exposed the relevance of FrC analysis regarding aging-related cognitive decline, it had two important limitations. First, even though fractal scaling exponents were found markedly different in the two age groups, no differences were found in broadband cross-spectral power—characterizing the strength but not the dynamics of coupling—and thus the plausible associations between connectivity strength and cognitive functions were not explored any further. Second, the analysis was carried out in a time-independent manner. Previous literature has proposed that regional neural activity as well as functional connections express not only scale-free, but *multifractal* dynamics [18, 27–29], suggesting that fractal scaling is a time-varying property. Therefore, in this study our main goal was to conduct a more detailed exploration of the relationship between FrC and impaired cognitive decline in healthy aging and alleviate these limitations, for a more detailed understanding of the relationship between FrC, fractal neural dynamics and cognitive decline in aging. Furthermore, FC strength and connectivity dynamics are more often found reduced in aging [7, 30–32]. It has been shown, however, that the presence of long-term autocorrelation can bias covariance estimation [33], and thus the known age-related reduction of fractal scaling in aging [23, 34] might affect connectivity estimates disregarding this property. Employing FrC analysis—in contrast to conventional FC approaches—can provide a way for addressing this notion.

Accordingly, we performed dynamic FrC (dFrC) analysis on electroencephalography (EEG) data collected from healthy young and elderly populations. To overcome the aforementioned limitations in the connectivity analyses of our previous study [26], we utilized detrended cross-correlation analysis (DCCA)—a method introduced by Podobnik and Stanley [35] to capture long-term coupling between non-stationary signals—to estimate dFrC. DCCA values were converted into the detrended cross-correlation coefficients (DCCC [36]), a measure more suitable for characterizing fractal connectivity strength in contrast to the theoretically unbounded DCCA. Univariate (i.e., regional) fractal scaling exponents of the analyzed signals were also estimated in a time-resolved manner, permitting a rough assessment of the strength of plausible multifractality of neural data in line with the so-called direct approach [37, 38]. After obtaining raw dFrC estimates, we utilized a machine learning approach based on recursive feature elimination to identify those connections that best discriminate between young and elderly individuals based on fractal connectivity strengths. We also compared mean and temporal variance of scaling exponents (i.e., mono- and multifractality of connections) between the two groups. Finally, we investigated the plausible relationships between FrC strength of most discriminative connections and performance measures from 7 different cognitive tests. Our results indicate that young and elderly individuals can be classified with high accuracy using FrC patterns which also show strong association to diminished cognitive performance in the elderly; however, the degree of multifractality appears similar in the two age groups.

## Materials and methods

### Participants and measurement protocol

In this study we analyzed the same dataset as in [26]. Therefore, here, we only provide a brief summary while for more details on the exclusion/exclusion criteria, measurement protocol and data curation steps, the reader is referred to our previous article. The final sample consisted of 43 healthy volunteers, with 24 assigned to the young (aged between 18 and 35 years, average: 25.37 ± 3.20) and 19 to the elderly (aged over 60 years, average: 66.39 ± 6.09) groups. None of the participants reported any history of a neuropsychiatric pathology or chronic pharmaceutical treatment that might affect their cognitive performance. Furthermore, they were instructed to avoid any substances affecting cognitive skills (e.g., alcohol, caffeine) for at least 3 h before the measurement and to have at least 6 h of sleep the preceding night. All participants provided written informed consent prior to the recordings and the study was approved by the regional ethics committee (Semmelweis University Regional and Institutional Committee of Science and Research Ethics, approval number: 2020/6).

The resting-state protocol consisted of a 3-min period, during which subjects were asked to stay idle but awake with eyes closed and refraining from any specific mental activity. Volunteers were first presented with an audio cue signaling the start of the measurement, while data collection itself started 5 s after to allow time for eye closure and for EEG to yield baseline activity. After 3 min the end of the recording was indicated by another auditory cue. Note that the full EEG measurement protocol then continued with a 3-min long eyes-open resting-state recording, and then three cognitive tests: an n-back working memory paradigm [39], a visual pattern recognition task [25] and a virtual spatial orientation task; however, evaluation of those datasets are beyond the scope of the current study. The entire EEG recording session took about 75 min, after which subjects proceeded to complete a set of standardized cognitive tests (see below).

### Detrended cross-correlation analysis

Dynamic fractal connectivity was assessed using the recently published real-time algorithm for DCCA (rtDCCA) computation [40]. Even though in this study we did not utilize online data analysis, the rtDCCA implementation provides a computationally efficient way of estimating DCCA (and DCCC) by greatly reducing processing time, and thus it is suitable for dynamic connectivity analysis. Please note that here we only provide a brief description of the method, while detailed descriptions are found in the original publications [35, 36, 40].

DCCA was originally developed for analyzing the covariance of non-stationary time series [35], such as EEG signals. This is achieved by locally detrending both signals—following integration—at a set of various scales s, computing the covariance of the residuals, then finally averaging the obtained values over the different analysis windows at the given scales used. Precisely, given two time series *x*_*t*_ and *y*_*t*_ of length *T* , their integrated versions *X*_*t*_ and *Y*_*t*_ are first obtained as follows:

1 $${\displaystyle \begin{array}{c}{X}_t=\sum\limits_{i=1}^t{x}_i\\ {}{Y}_t=\sum\limits_{i=1}^t{y}_i.\end{array}}$$

Then, at given scale *s* the signals are divided into *Ks* = *T* − *s* + 1 overlapping windows (each of length *s*). In each window *k* the local trends ${\overline{X}}_k$ and ${\overline{Y}}_k$ are obtained via ordinary least squares (OLS) regression and removed from the signals, and the covariance of residuals is computed as follows [41]:

2 $${f}_{DCCA}^2\left(s,k\right)=\frac{1}{s-1} \sum\limits_{k=i}^{i+s}\left({X}_i-{\overline{X}}_{k,i}\right)\left({Y}_i-{\overline{Y}}_{k,i}\right).$$

Finally, ${F}_{DCCA}^2(s)$ is obtained via averaging over all windows *k*:

3 $${F}_{DCCA}^2(s)=\frac{1}{K_s-1}{\sum}_{k=1}^{K_s}{f}_{DCCA}^2\left(s,k\right).$$

Notably, the procedure can be equivalently performed by using non-overlapping windows of length *s*. Also, in the case of *x*_*t*_ = *y*_*t*_, the formula reduces to that of Detrended Fluctuation Analysis (DFA), allowing for obtaining the fractal scaling exponent of univariate signals [42]. Therefore, by utilizing the matrix notation introduced Kaposzta, Czoch [40], both DCCA and DFA scaling functions (and thus coefficients) of multivariate signals can be obtained at the same time in an efficient manner.

DCCA in of itself, however, is not necessarily a suitable measure for connectivity analyses, as the obtained values are theoretically unbounded, and thus DCCA is rather utilized to obtain the bivariate fractal scaling exponent of long-term coupled processes [41]. However, DCCA can be further developed into the detrended-cross correlation coefficient at scale *s* (*DCCC*(*s*)) by dividing the obtained bivariate scaling function ${F}_{DCCA}^2(s)$ [36]:

4 $$DCCC(s)=\frac{F_{DCCA}^2(s)}{\sqrt{F_{DFA.X}^2(s)\cdot {F}_{DFA,Y}^2(s)}},$$

where ${F}_{DFA.X}^2(s)$ and ${F}_{DFA.Y}^2(s)$ are obtained by performing the procedures described in Eqs. (1)–(3) with making *y*_*t*_ = *x*_*t*_ and *x*_*t*_ = *y*_*t*_, respectively. It can be shown that this measure is indeed bounded between –1 and 1 and therefore can be utilized to estimate the strength of functional coupling between two non-stationary processes [41]. Furthermore, as the analysis is carried out over multiple time scales, computing DCCC between a set of EEG signals yields a set of connectivity matrices, one for each scale s of the analysis. Finally, in the rtDCCA setting the analysis is performed in a sliding window fashion, thus providing a three-dimensional connectivity matrix of shape *N*_*c*_ · *N*_*c*_ · *N*_*s*_—where *N*_*c*_ and *N*_*s*_are the number of EEG channels and the number of applied scales, respectively—for every time point.

To obtain a robust characterization of functional connectivity, we carried out rtDCCC analysis with the following parameters. The number of scales was set to 5, ranging from 2^3^ to 2^7^ data points in a dyadic manner. Note that these scales roughly correspond to fluctuations in the frequency range of 2 to 32 Hz. The sliding window size was set to 2 s of data (1024 data points), with a step size of 0.5 s (128 data points, 75% overlapping windows). This procedure yielded *N*_*t*_ = 137, 3-dimensional DCCC matrices for each subject. Additionally, univariate DFA-exponents were computed in every window via OLS regression of DFA scaling function value on scale following log-log transformation.

### Cognitive battery

Cognitive performance was assessed via the Cambridge Neurophysiological Test Automated Battery (CANTAB), a state-of-the-art, standardized and validated tool for cognitive neuroscience (ref?). CANTAB comprises of a collection of short (i.e., few minutes-long) tests, each designed to assess various aspects of cognition, such as visual information processing or spatial/temporal working memory. In this study we employed 7 tests (see below). These were selected as they challenge cognitive domains that are commonly affected in age-related cognitive decline and even in early stages of dementia, as well as this set of tests was found sensitive in revealing differences in cognitive performance between young and elderly cohorts by previous studies [3, 26]. Participants used a 10.2-in Apple iPad (9th generation) to complete the battery. Participants were provided with a digital booklet prior to measurement detailing task instructions, as well as each task started with a short introduction and practice session for the given test (in a language the user was comfortable with), allowing the participants to complete the session alone in an isolated room excluding external influence/help from the experimenter. The cognitive assessment session lasted for about 30–50 min, depending on performance. Users were instructed to complete the tasks using the index finger of their dominant hand.

The seven cognitive tasks were as follows: (*I*) first, the *motor screening* task (MOT) was presented, during which the user had to tap on various colored crosses appearing at changing locations as fast and accurately as possible (output measures: reaction time, precision). (*II*) Subsequently, *reaction time* (RTI) was assessed in a manner where a changing number (one or five) of circles were presented on the top of the screen with a button on the bottom. The user had to hold their finger on the start button until one of the circles turned yellow, after which they had to release and quickly tap on the target circle (output measures: release time, movement time, accuracy). (*III*) *Paired associates learning* (PAL) was tested with boxes being displayed on the periphery of the screen, each of which presenting a unique pattern in a randomized order. Then, each pattern was consecutively projected to the middle of the display, and the volunteer had to correctly identify the box which originally included the currently presented pattern. In case of an error, the boxes again showed each pattern before restarting the trial, with the pattern locations staying fixed (output measures: number of errors, number of attempts, first attempt memory score). (*IV*) *Pattern recognition memory* (PRM) was assessed by first presenting the participant with a series of abstract visual patterns, and then in a later phase (~15-min delay, i.e., after completing subsequent test), asking them to correctly discriminate between a pattern they were already presented before and a novel pattern appearing side by side on the screen (output measures: response latency, number and percentage of correct responses). (*V*) During the latency period needed for the PRM test, *spatial working memory* (SWM) was assessed via colored boxes scattered around the screen. Selecting boxes randomly via a process of elimination, the participant had to find a yellow token hidden under one of the boxes. When found, another token was placed, always under a different box. The task ended when a token was found for all boxes (output measures: between errors, within errors, total errors, search strategy). (*VI*) *Rapid visual processing* (RVP) was tested with a central white box that displayed digits from 2 to 9, appearing individually in a pseudo-random order at 100 digits per minute. Volunteers are tasked with identifying 1 to 3-digit sequences (e.g.: 2, 4-6, 3-5-7) and reacting to a positive match with a press of a button located below the square as fast as possible (output measures: response latency, number of correct responses, percentage of correct responses). Finally, (*VII*) *delayed matching to sample* (DMS) was employed with presenting a complex visual pattern followed by the original and three similar but slightly different patterns, after a brief delay (0, 4, or 12 s). The participants were tasked with selecting the originally presented pattern as quickly as possible (output measures: response latency, number of correct responses).

For more details and visual illustrations of the selected cognitive tasks, the reader is referred to the official CANTAB website (https://www.cambridgecognition.com/cantab/).

### EEG data acquisition and pre-processing

Measurements took place in a dark, electrically shielded room where the participants were asked to sit in a comfortable office chair facing a 24-in computer display and a small speaker. Data acquisition was carried out using an Emotiv Epoc+ wireless EEG system (Emotiv Systems Inc., San Francisco, CA, USA) and its proprietary data management software (EmotivPRO, Emotiv Systems Inc., San Francisco, CA, USA). This setup allowed for the capturing of neural activity from 14 standard locations in accordance with the international 10-10 system (AF3, AF4, F3, F4, F7, F8, FC5, FC6, T7, T8, P7, P8, O1, and O2) with CMS and DRL references at P3 and P4 and ground electrodes positioned at the left and right mastoid processes. The device sampled neural activity at an internal frequency of 2048 Hz, which was then passed through a 5th order digital Sinc filter with cutoff frequencies 0.2 and 45 Hz and notch filters at 50 and 60 Hz before down-sampling to 256 Hz and transmitting to the recording computer. Electrode impedance was kept below 20 kΩ so that all measurements could be carried out with maximal contact quality as per manufacturer recommendations.

Raw EEG data was first passed through an additional 4th order, zero-phase Butterworth filter to attenuate effects of spike-like artifacts and slow drift (cutoff frequencies: 0.5 and 45 Hz). Then, data was visually inspected by two experimenters to ensure signal quality was appropriate, and artifact-free epochs were identified and selected from every recording, keeping only those segments that were considered artifact-free by both experimenters independently. Finally, to ensure equal amount of data from each subject, epochs were trimmed randomly to the length of the shortest obtained epoch, resulting in a final length of 72 s of EEG data for all subjects. All following pre-processing steps were carried out in MATLAB (MathWorks, Natick, MA, USA) using custom functions and scripts or ones of the EEGLAB toolbox [43]. Artifact removal was carried out via utilizing independent component analysis (ICA). In that, data was decomposed into linearly independent components using the infomax algorithm [44]. Components were visually inspected by two investigators adept in EEG analysis, and those associated with eye movements, skeletal muscle activity, or other sources of noise (such as heart activity or head motion) were identified based on their spectral, spatial, and time-domain features [45, 46] and rejected before reverse ICA transformation. Finally, the cleansed EEG epochs were re-referenced to the common average reference. For more details on data selection and pre-processing, please see Czoch, Kaposzta [26].

### Selection of most discriminative connectivity features

One of our main goals was to test the power of dynamic fractal connectivity measures to distinguish between young and elderly participants. The previously described analysis yielded a set of 137 time points * 14*(14−1)/2 channel pairs * 5 scales = 62335 features for each subject. Therefore, we applied a feature selection method termed Support Vector Machine with Recursive Feature Elimination (SVM-RFE [47]) to select those variables that are most discriminative between young and elderly subjects. We chose to utilize SVM-RFE as it has been proven effective in identifying connectivity patterns discriminating between various cognitive states [48]. A brief description of SVM-RFE is provided here, while more details of the procedure can be found in the original article of Yan and Zhang [47]. SVM-RFE eliminates irrelevant features in a recursive fashion as follows. The dataset is first divided into training and tests sets. Then, at every iteration, a support vector machine (SVM) is trained on a training set and evaluated on test set data. Feature importances are extracted as the square of support vector weights, and a pre-defined proportion of least important features (as ranked by their weights) are discarded from the feature set. To avoid removing correlated features collectively identified as less relevant, we also employed the correlation bias reduction (CBR) step proposed in [47], where at the end of every iteration, if a cluster of highly correlated features were detected in the set of eliminated features, the one with the highest importance was restored to the remaining active features (see below). In the next iteration, another SVM is trained on the now-reduced dataset, and features are recursively using the procedure described above, until only one feature remains. After the procedure is finished, features are ranked in order of their removal. Finally, the set of most discriminative features is obtained in another recursive procedure, when features are added back one by one in decreasing order of importance, and at each iteration an SVM is trained on the training set and evaluated on the test set. The feature set yielding the best classification accuracy is denoted as the set of most discriminative features between young and elderly groups.

SVM-RFE was performed following the guidelines proposed in [47] and the pipeline adopted for EEG connectivity features presented in [48]. In case of large feature sets many features can be highly correlated—indicating that they effectively capture the same information—, which can lead to correlation bias as their importance can be wrongly underestimated [49, 50]. This also holds for connectivity features, and hence SVM-RFE should be carried out incorporating the CBR step. In this approach, if there are highly correlated clusters of features in the eliminated batch, a representative feature per cluster (the one with the highest importance) can be added back to the retained features. For computational efficiency, in each iteration 50% of features were eliminated, and then representative features were added back to reduce correlation bias in case of highly correlated features in the removed batch. The threshold for identifying such clusters were set to 0.9 as suggested in [47]. The procedure was continued until only 20 features remained, from when on features were removed one by one. In order to use information in the data most efficiently, SVM-RFE was carried out in an extensive leave-one-subject-out cross-validation (ELOO-CV). Precisely, in each ELOO-CV iteration, one subject was selected from each group to form the test dataset, and SVM-RFE was performed with the rest of the data used as training set. This way if splitting the data is essential to ensure that classification is based on group effect and not on unique individual patterns. The procedure was extensive, as all possible train-test divisions (24 · 19 = 456) were utilized, and final feature rankings were obtained as the average taken over ELOO-CV iterations. Finally, following SVM-RFE the selection of the set of most discriminative features was also performed using ELOO-CV, where the most discriminative set was defined as the one producing the highest average ELOO-CV accuracy. During SCM-RFE, SVMs with a linear kernel were utilized. The optimal choice of the SVM regularization parameter was obtained by carrying out the entire procedure using a range of values (10^−2^, 10^−1^, 1, 10, and 100).

Performance was characterized on two levels. First, we computed sample-wise accuracy (the proportion of correctly classified examples to all examples) in each iteration of the ELOO-CV, i.e., each test example denoting the connectivity state of a test subject at a given time was classified as coming from either a young or an elderly individual. Sample-wise accuracies were averaged over ELOO-CV iterations and contrasted with chance level obtained at *α* = 0.0001 with assuming a binomial distribution of classification errors [51]. We evaluated accuracy on the subject level, too. In that, at every iteration of the ELOO-CV, a majority vote was taken over all predictions for the examples of a given test subject, producing the subject-wise group label prediction. Subject-wise accuracies were then computed and averaged over all ELOO-CV iterations. Additionally, Cohen’s Kappa values were also computed in both settings, providing a more explicit measure if classification performance surpasses chance level [52].

### Statistical analyses

Output variables from CANTAB evaluation were compared between young and old groups with two-sample t-tests or Mann-Whitney U tests, depending on the normality of the datasets as evaluated with Lilliefors tests. The obtained *p*-values were adjusted for multiple comparisons using the false discovery rate (FDR) procedure by Benjamini and Hochberg [53]. Note that this analysis was carried out previously and reported in [26].

Performance of group separability was assessed as described previously. We also performed post hoc analyses of dFrC measures to better understand the nature of differences between the two age groups. In that, mean (*μ*_*DCCC*(*s*)_) and variance (${\sigma}_{DCCC(s)}^2$) of all connections at all scales were computed in the set of connections selected by the SVM-RFE method. Since these tests were performed on a pre-selected sample of data (and thus these comparisons were only conducted to better capture the nature of between-group differences), we did not adjust for multiple comparisons. Additionally, mean and variance of DFA-exponents for all 14 brain regions were computed and contrasted between the two groups using two sample t tests or Mann Whitney U tests depending on normality of data. Results were adjusted for multiple comparisons via FDR, given that DFA-exponents were not included in the SVM-RFE model.

The other major goal of this study was to explore if dynamic fractal connectivity features identified as the most discriminative between young and elderly groups are associated with cognitive performance as assessed via CANTAB. In that, for each subject the mean and variance of fractal connectivity over time was computed at every scale for every connection, then for each feature the Spearman correlation coefficient was computed with all CANTAB output measures that were found different between the young and elderly groups. Given that this analysis was carried out on an already curated dataset (involving only those connections that were identified as discriminative, as well as only CANTAB measures significantly differing between the two age groups), the *p*-values obtained for the Spearman correlation coefficients were not adjusted.

## Results

### Cognitive scores

Results of this analysis have been reported in detail in [26]; therefore, here, we only provide a brief summary. In terms of the motor function assessing MOT task, our analysis did not reveal any significant differences in either of the recorded metrics. This finding decreases the likelihood that the differences observed in other tasks are attributable to sensorimotor impairments or decline, and instead suggests that they are more plausibly associated with cognitive differences between the two groups. In addition, overall completion time of the cognitive battery was significantly longer for the elderly group (53.37 vs. 57.92 minutes for young and elderly, respectively, Wilcoxon rank-sum test *p* < 0.0001). For the remaining 6 tests, we found significant between-group differences in CANTAB output measures in 54 cases: 16 for PAL, 12 for SWM, 10 for DMS, 8 for RVP, and 4-4 for RTI and PRM tasks. In general, individuals in the elderly group executed time-sensitive tasks (i.e., when a response had to be provided as fast as possible) with increased latency when compared to those in the young group, shown in outcomes of 4 cognitive tests (i.e., DMS, PRM, RTI, and RVP). Cognitive performance (as most often captured in response accuracy, or the number of correct/erroneous responses) was also found decreased in the elderly group in 4 out of 6 tasks (PAL, RTI, RVP, and SWM), noting that in the RTI and RVP tests both response time and performance was found negatively affected in aging. A more detailed description of these outcomes is found in [26] as well as all significant differences are presented in Supplementary Table S1.

### Discriminating fractal connectivity between young and elderly

When selecting the optimal model to perform classification, we tested a set of different values for the regularization parameter *C*, since its optimal value depend on the given dataset and is most commonly chosen on a case-by-case basis. In fact, the regularization parameter *C* is the inverse of the regularization constant *λ*. A higher value of (and thus a lower value of *C*) puts more weight to the regularization term in the SVM cost function [54] and thus promotes large margin classification (i.e., less overfitting) and vice versa. As it was not known in advance how well the data was linearly separable, we chose to iterate through powers of ten ranging from –2 to 2, in order to cover a wide range of regularization parameter values. It is important to note that setting *C* too high or too low may introduce overfitting or underfitting, respectively, along with numerous other unintended behaviors of the model such as vastly increased training time, model instability or high bias [55].

We obtained the best overall results by setting *C* = 10^−2^; however, final cross-validation accuracies for other values of *C* were also comparable (see Table 1). This model identified 56 connections (from all 5 temporal scales considered) as most discriminatory between young and elderly individuals. When using this selective feature set, the model achieved an average sample-wise ELOO-CV accuracy of 89.48% with a Cohen’s Kappa coefficient of 0.7896. This indicated a significantly better than chance performance, which was found to be 61.31%. As expected, subject-level performance was even superior with 92.11% accuracy and a Kappa value of 0.8421. These results suggested that the SVM-RFE indeed identified functional connections that reliably discriminated between young and elderly individuals.

**Table 1:** Performance measures for all values of regularization parameter *C*. *Acc*_*SW*_: sample-wise accuracy during cross-validation; *Kappa*_*SW*_: sample-wise Cohen’s Kappa value during cross-validation; *Acc*_*subj*_: subject-wise accuracy during cross-validation; *Kappa*_*subj*_: subject-wise Cohen’s Kappa during cross-validation

*C*	# connections	*Acc*_*SW*_	*Kappa*_*SW*_	*Acc*_*subj*_	*Kappa*_*subj*_
10^−2^	56	89.48%	0.7896	92.11%	0.8421
10^−1^	90	87.07%	0.7414	87.72%	0.7544
1	113	89.28%	0.7856	92.76%	0.8553
10	112	89.02%	0.7804	92.11%	0.8421
100	112	89.02%	0.7804	92.11%	0.8421

**Fig. 1:**
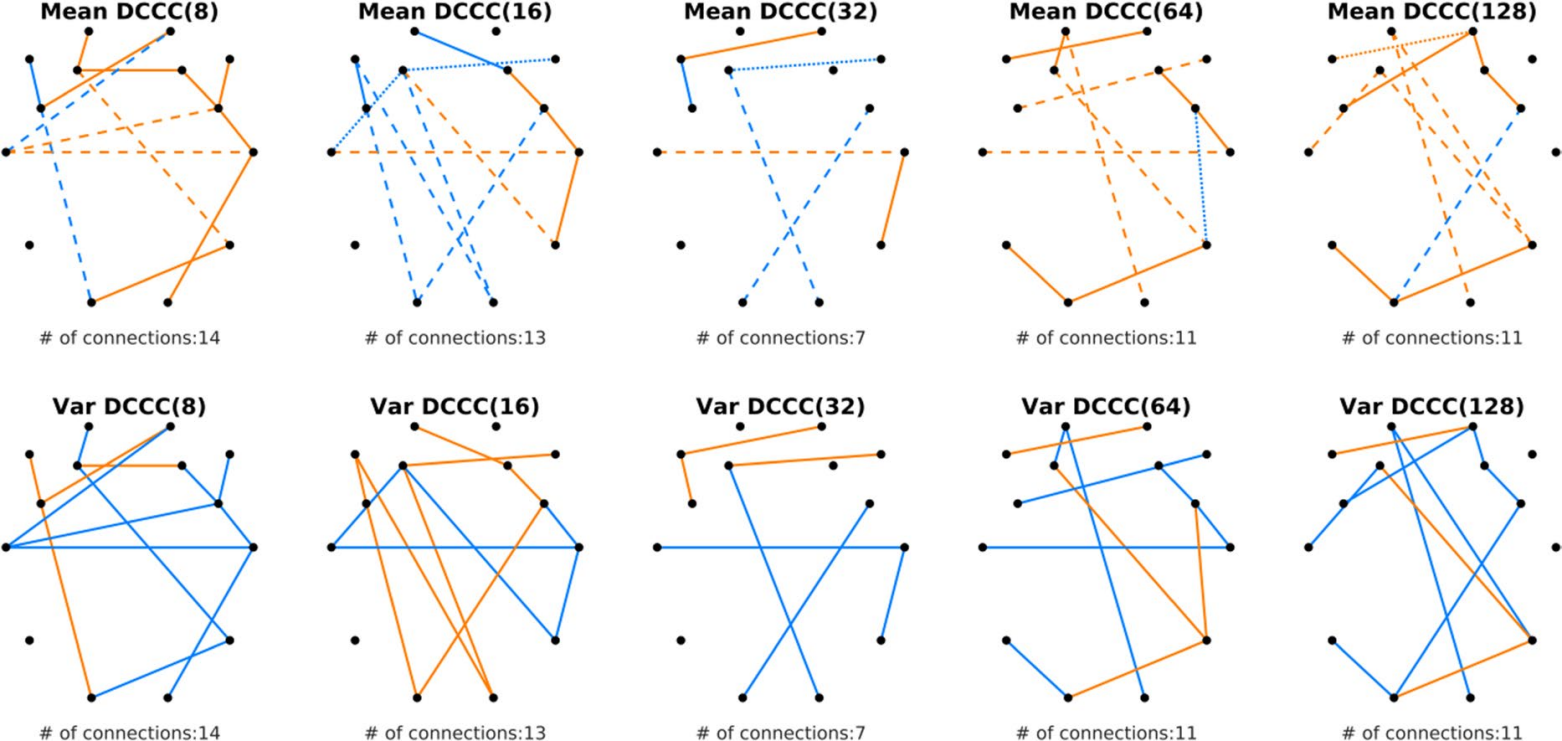
Most discriminative connections between young and elderly groups across the five time scales investigated (columns). Black dots denote the 14 EEG channels with their layout reflecting the utilized montage from a top-down view, while colored lines represent the connections identified by SVM-RFE linking the corresponding brain regions. Rows show how *μ*_*DCCC*(*s*)_ (upper) and ${\sigma}_{DCCC(s)}^2$ (lower) differed in the two groups, with orange and blue indicating higher absolute values in the elderly and in the young groups, respectively. In the upper row, solid/dashed lines denote connections with positive/negative grand averages in both groups, while dotted lines indicate that the sign of *μ*_*DCCC*(*s*)_ was opposite in the two age groups

Topology and nature of the 56 most discriminatory connections are illustrated in Fig. 1. In general, connections were selected from over the entire cortex, as well as the 56 connections were distributed approximately evenly among the 5 different time scales, indicating age-related alterations of neural dynamics in a broad range of brain rhythms. Proportion of connections with positive and negative *μ*_*DCCC*(*s*)_ values were fairly balanced in both groups (32 to 24 in the young and 29 to 27 in the elderly group). Notably, the strength of FrC—i.e., the absolute value of *μ*_*DCCC*(*s*)_ including both positive and negative connections—tended to be higher in the elderly group (41 out of 56 connections overall), except at *s* = 16 where the pattern was rather dominated by more negative occipito-frontal connections in the young group. Additionally, only 5 connections were among the set where the sign of *μ*_*DCCC*(*s*)_ was opposite in the two age groups. On the other hand, when investigating the variance of the identified connections, it appeared that ${\sigma}_{DCCC(s)}^2$ was higher in the young when compared to the elderly group in the majority (34 out of 56) of cases. Again, in contrast to the general pattern, at *s* = 16 occipito-frontal connections could be characterized with higher ${\sigma}_{DCCC(16)}^2$ in the elderly than in the young group.

As noted previously, conventional statistical analysis would have been impractical in case of such a wide set of variables (455 connections disregarding time, at sample sizes 24 and 19), hence necessitating the implementation of the feature selection approach. However, to gain more insight on the nature of dFrC differences between young and elderly populations, we compared *μ*_*DCCC*(*s*)_ and ${\sigma}_{DCCC(s)}^2$ of the selected 56 connections between the two groups. Results of these post hoc analyses are presented in Table 2. Precisely, *μ*_*DCCC*(*s*)_ was found significantly different (*p* < 0.05, unadjusted) in case of 9 connections. Notably, 7 out of 9 indicated anticorrelated activity between the given brain region pairs in both groups, with the elderly population expressing more negative values, while for the remaining 2 connections, positive connection strength was also weaker in the young group. On the other hand, ${\sigma}_{DCCC(s)}^2$ was found differing for only 2 connections, showing decreased dFrC variance in the elderly.

**Table 2:** Connections among the selected 56 channels showing difference between young and elderly groups in *μ*_*DCCC*(*s*)_ and ${\sigma}_{DCCC(s)}^2$. *Ch*_1, 2_: corresponding channels of the given connection; *s* : temporal scale of DCCC analysis; *E*_*young*, *elderly*_: the expected value of the given measure in the young and elderly groups, respectively

Measure	*Ch*_1_	*Ch*_2_	*s*	*E*_*young*_	*E*_*elderly*_	*p*
*μ*_*DCCC*(*s*)_	AF3	F3	8	0.4773	0.6286	0.0425
	T7	T8	16	−0.2775	−0.3924	0.0388
	T7	T8	32	−0.2696	−0.3849	0.0222
	AF3	F3	64	0.4082	0.5876	0.0287
	AF3	O2	64	−0.5464	−0.6683	0.0490
	F3	P8	64	−0.4501	−0.5851	0.0114
	T7	T8	64	−0.1623	−0.3212	0.0411
	AF3	O2	128	−0.4959	−0.6339	0.0237
	F3	P8	128	−0.4053	−0.5587	0.0064
${\sigma}_{DCCC(s)}^2$	T7	T8	8	0.0103	0.0074	0.0324
	T8	FC6	64	0.0241	0.0161	0.0396

**Fig. 2:**
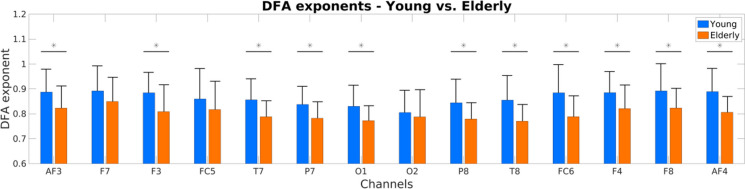
DFA scaling exponents in the young (blue) and elderly (orange) groups. Asterisk symbols denote significant differences (*p* < 0.05, FDR-adjusted) between the two age groups

Finally, we evaluated the mean and variance of regional DFA-exponents (Fig. 2). Note that the analysis regarding the mean values is equivalent to that carried out in [26], given that scaling exponents obtained in the frequency and time domains are inherently related [56, 57] and thus we expected similar results. Indeed, we found reduced mean DFA-exponents in the elderly group over 11 cortical regions (AF3, AF4, F3, F4, F8, FC6, T7, T8, P7, P8, and O1, *p<*0.05 in all cases, FDR-adjusted) when compared to those in the young group. However, no significant between-group differences could be identified in the temporal variance of fractal scaling (i.e., degree of multifractality).

## Task specific topology

The applied cognitive battery encompassed a diverse set of tests designed to assess various aspects of cognitive functioning. Since different cognitive functions could be associated to a varying set of brain regions [58], it is crucial to evaluate which connections may be associated with performance in specific cognitive domains. Hence, we present the topology of significant correlations found between dFrC indices (*μ*_*DCCC*(*s*)_ ${\sigma}_{DCCC(s)}^2$) and cognitive performance measures for each CANTAB task individually. On all figures, lighter/darker shades indicate a negative/positive correlation with the given cognitive measure, with continuous lines denoting connections correlated with accuracy (ACC)-like measures (e.g., number of correct responses/errors), dashed lines indicating those associated to response time (RT)-like measures (e.g., response latency), and dotted lines those that were found related to both cognitive dimensions. For the sake of clarity, please note that this notation always reflects relation to ideal task performance and not the sign of the correlation coefficient itself between dFrC measures and cognitive scores; i.e., a dark blue line implying po*μ*_*DCCC*(*s*)_ and RT*μ*_*DCCC*(*s*)_ and response latency, where shorter latency means better performance (and similarly for positive/negative correlations with number of correct responses/errors, respectively). A summary of identified correlations between dFrC and CANTAB scores in all tasks are provided in Table 3, while below we present task-specific topologies for each test individually.

**Table 3:** Total number of correlations found between dFrC and fractal neural dynamics and CANTAB output measures

		DMS	PRM	RVP	RTI	PAL	SWM
Young	*μ*_*DCCC*(*S*)_	19	3	10	19	19	8
	${\sigma}_{DCCC(s)}^2$	26	7	16	11	32	17
	*μ*_*DFA*_	3	0	6	0	0	0
Elderly	*μ*_*DCCC*(*S*)_	31	11	9	12	20	31
	${\sigma}_{DCCC(s)}^2$	39	6	4	4	48	29
	*μ*_*DFA*_	0	2	20	6	7	0

**Fig. 3:**
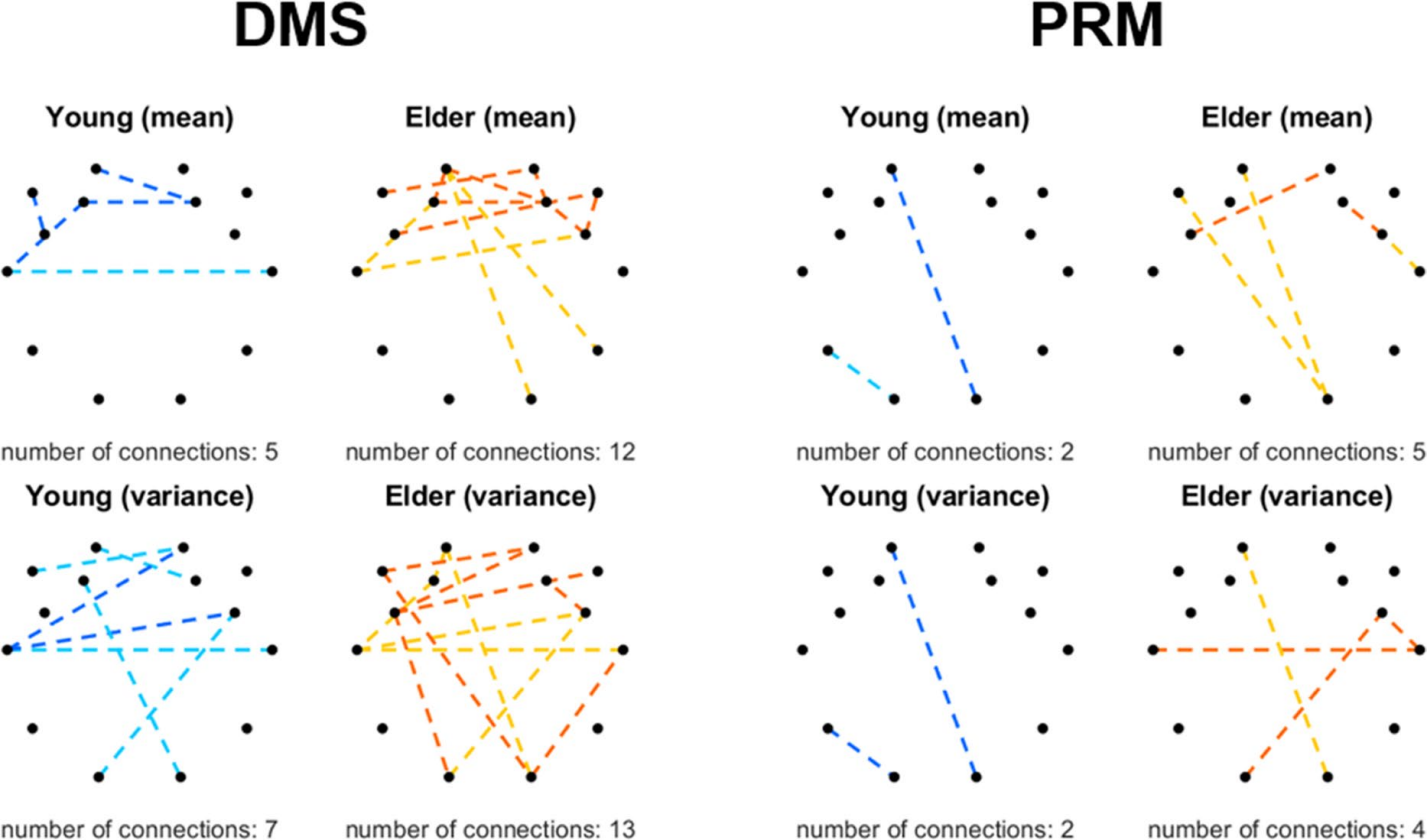
Connections expressing correlations with output measures in the DMS (left) and PRM (right) tasks. Black dots denote the 14 EEG channels with their layout reflecting the utilized montage from a top-down view, while colored lines represent the connections linking the corresponding brain regions where a significant relationship was found between connectivity and behavior in at least one of the five temporal scales. Young and elderly groups are illustrated using blue and orange colors, respectively. Positive correlations are marked with a darker color, and negative correlations are marked with a lighter color. Dashed and solid lines represent temporal and accuracy-based metrics, respectively. Upper row denotes correlations with *μ*_*DCCC*(*s*)_, while the lower row is the same for ${\sigma}_{DCCC(s)}^2$

Visual pattern recognition memory and matching ability was challenged by the DMS and PRM tasks, where performance was characterized by RT and ACC (Fig. 1). Notably, dFrC was only found associated with RT but not with ACC in both tasks. Precisely, response latency in the DMS task had an overall distinct association with frontal intra- and inter-hemispheric patterns with a left hemispheric dominance involving inter-parietal connections in the young group (Fig. 3, left). This was apparent in both mean and variance of *DCCC*(*s*), with the latter also involving occipitofrontal connections. Conversely, the elderly group exhibited more complex intra- and inter-hemispheric associations between *μ*_*DCCC*(*s*)_ and RT, generally involving most of the frontal and prefrontal regions. DMS RT indices were found correlated with ${\sigma}_{DCCC(s)}^2$ of connections spanning the whole cortex, however substantially more connections were found related to DMS performance in the elderly (13) than in the young (7) group. In both age groups, dFrC indices of most frontal connections were positively related to response time, while most longitudinal (e.g., occipito-frontal) connections expressed an inverse relationship with performance. On the other hand, PRM expressed remarkably few associations with dFrC in contrast to the quite similar DMS task, mostly involving occipito-frontal connections (Fig. 3, right). Similar to DMS, however, dFrC was correlated with RT measures for more connections in the elderly than in the young group in terms of both mean and variance of *DCCC*(*s*) and most longitudinal connections were anticorrelated to task performance.

**Fig. 4:**
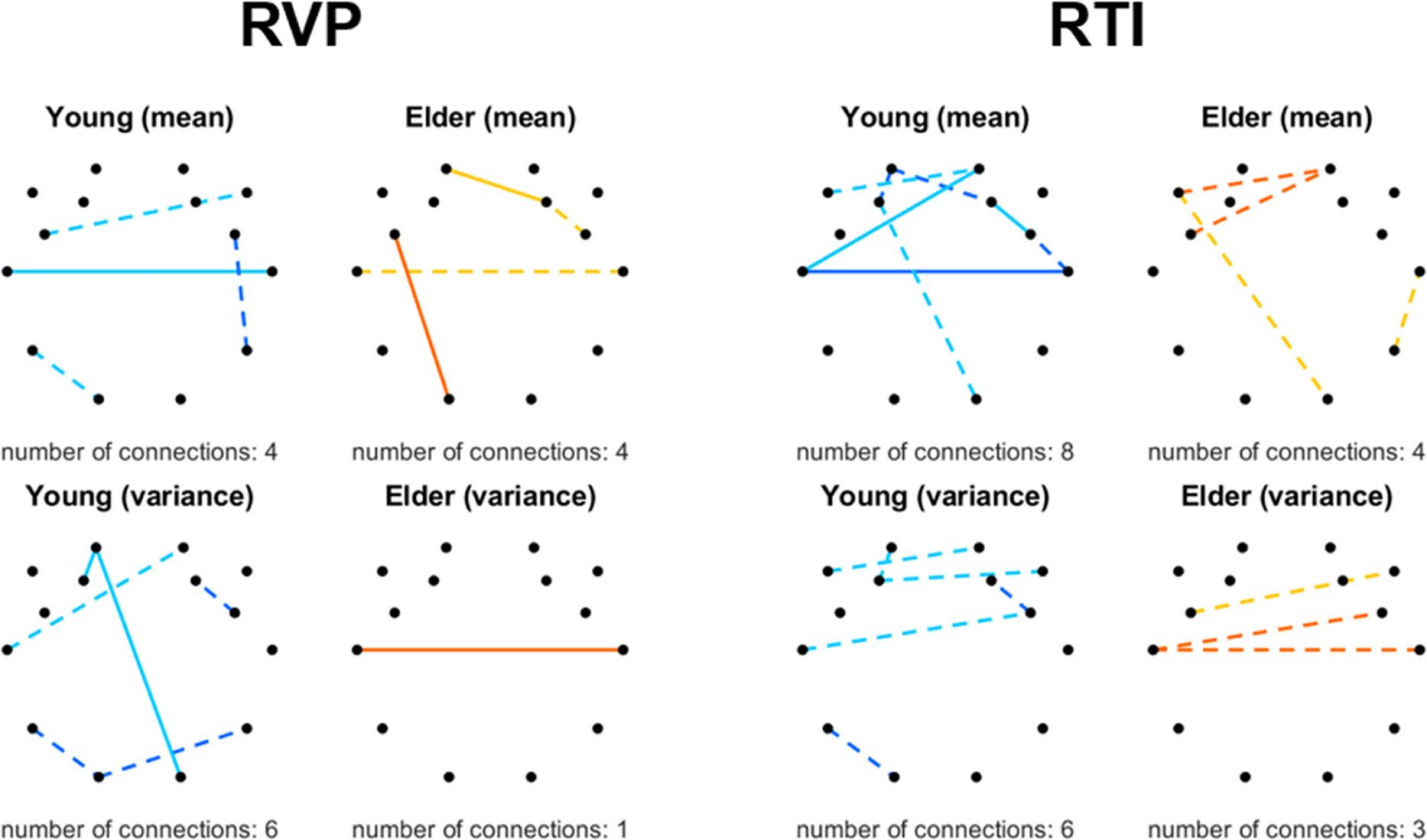
Connections expressing correlations with output measures in the RVP (left) and RTI (right) tasks. Black dots denote the 14 EEG channels with their layout reflecting the utilized montage from a top-down view, while colored lines represent the connections linking the corresponding brain regions where a significant relationship was found between connectivity and behavior in at least one of the five temporal scales. Young and elderly groups are illustrated using blue and orange colors, respectively. Positive correlations are marked with a darker color, and negative correlations are marked with a lighter color. Dashed and solid lines represent temporal and accuracy-based metrics, respectively. Upper ${\mu}_{DCCC(s)}\ {\sigma}_{DCCC(s)}^2$

Counter to the previous tasks, RVP and RTI tested other cognitive domains, namely, sustained attention, and motor and mental response speed. Connections that correlated with CANTAB measures (for both RT and ACC) are illustrated in Fig. 4. In both tasks, most dFrC indices were found correlated to RT; however, some were also identified sporadically as being related to task ACC. Interestingly, none of the connections expressed a significant correlation with RT and ACC simultaneously. Notably, very few longitudinal connections were found related to task performance. In detail, RVP performance only expressed sporadic associations with dFrC indices, with a tendency towards interhemispheric connections (Fig. 4, left). With regards to RTI, the spatial pattern was found similar to that with RVP, only more connections were identified as related (involving as well *μ*_*DCCC*(*s*)_ of that linking O2 and F3 or F7) with a frontal dominance (Fig. 4, right). Also, in sharp contrast with DMS and PRM, more connections were found correlated to task performance in the young compared to the elderly group in case of RVP and RTI tasks.

**Fig. 5:**
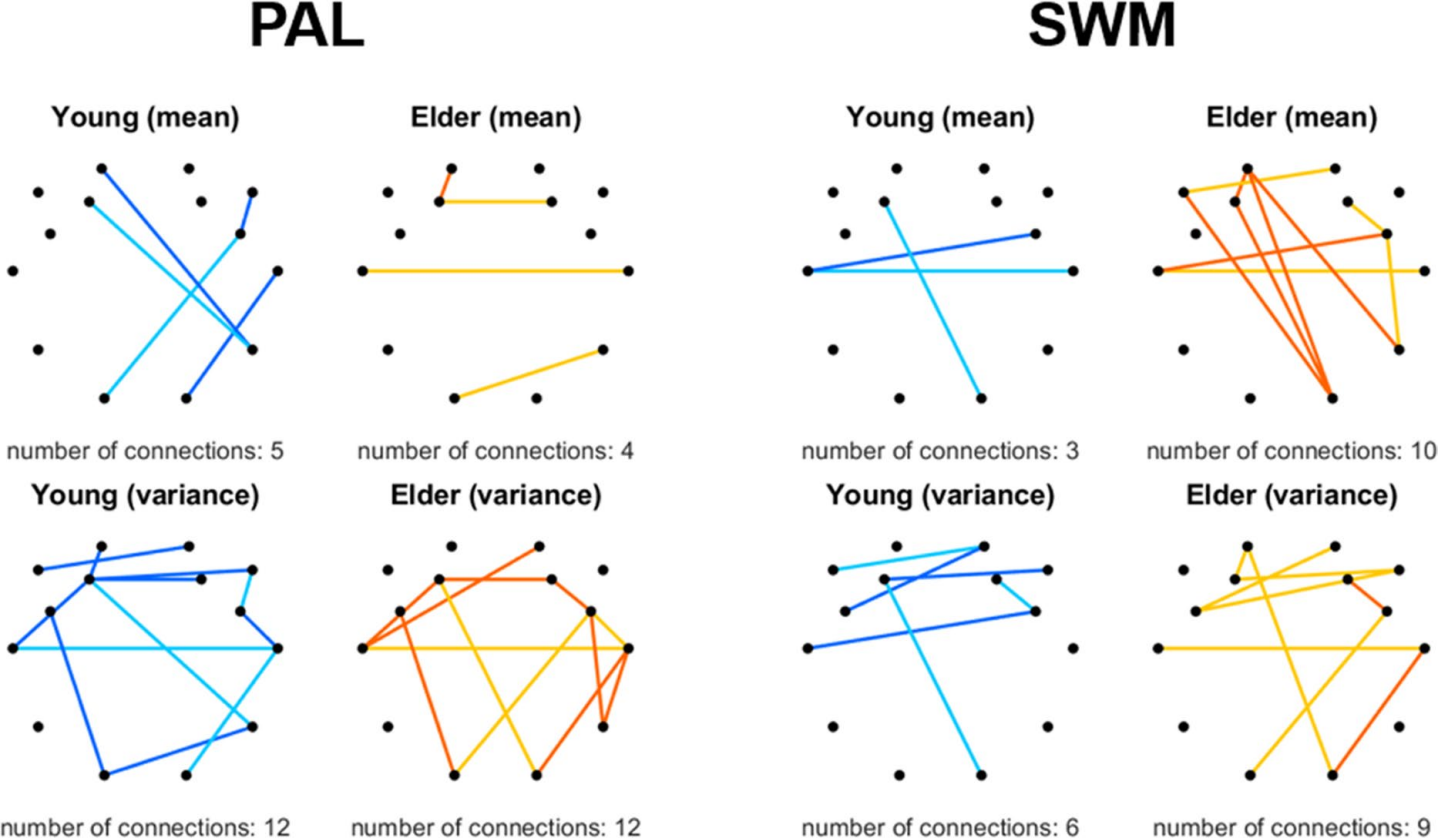
Connections expressing correlations with output measures in the DMS (left) and PRM (right) tasks. Black dots denote the 14 EEG channels with their layout reflecting the utilized montage from a top-down view, while colored lines represent the connections linking the corresponding brain regions where a significant relationship was found between connectivity and behavior in at least one of the five temporal scales. Young and elderly groups are illustrated using blue and orange colors, respectively. Positive correlations are marked with a darker, negative correlations with a lighter color. Dashed and solid lines represent temporal and accuracy-based metrics, respectively. Upper ${\mu}_{DCCC(s)}\ {\sigma}_{DCCC(s)}^2$

Finally, PAL and SWM tasks required memorizing and manipulating visuo-spatial information (Fig. 5). Response time was not considered in these tasks but instead performance was characterized by task accuracy and strategy. Additionally, these tasks were more complex and difficult than those discussed previously. The PAL task, where most significant differences in task performance (for 16 CANTAB measures) were found, showed to be associated with a more inter-hemispheric pattern (Fig. 5, left). In the elderly population this concerned identical regions of the different hemispheres for *μ*_*DCCC*(*s*)_, whereas in the young cohort *μ*_*DCCC*(*s*)_ of occipito- and parieto-frontal connections was found correlated with PAL performance. Variance of *DCCC*(*s*) in both groups exerted a global association pattern, which, however, showed less involvement in the frontal lobe. Notably, this topology was found comparable in the two age groups. Finally, SWM performance showed an association pattern to dFrC quite similar to that found in case of DMS (Fig. 5, right). In that, in terms of both *μ*_*DCCC*(*s*)_ and ${\sigma}_{DCCC(s)}^2$, dFrC was found correlated with task performance for fewer connections in the young compared to the elderly group. Also, these involved mostly frontal regions in the young cohort, while expressed a global pattern in elderly individuals.

It is important to stress that Figs. 3, 4, and 5 only illustrate a summary of the patterns for the various tasks, i.e., results were collapsed in case of all scales and all CANTAB output variables for the given tasks. Therefore, we discuss task performance measures associated with dFrC in more detail below.

### Correlation of cognitive scores with fractal connectivity measures

**Fig. 6:**
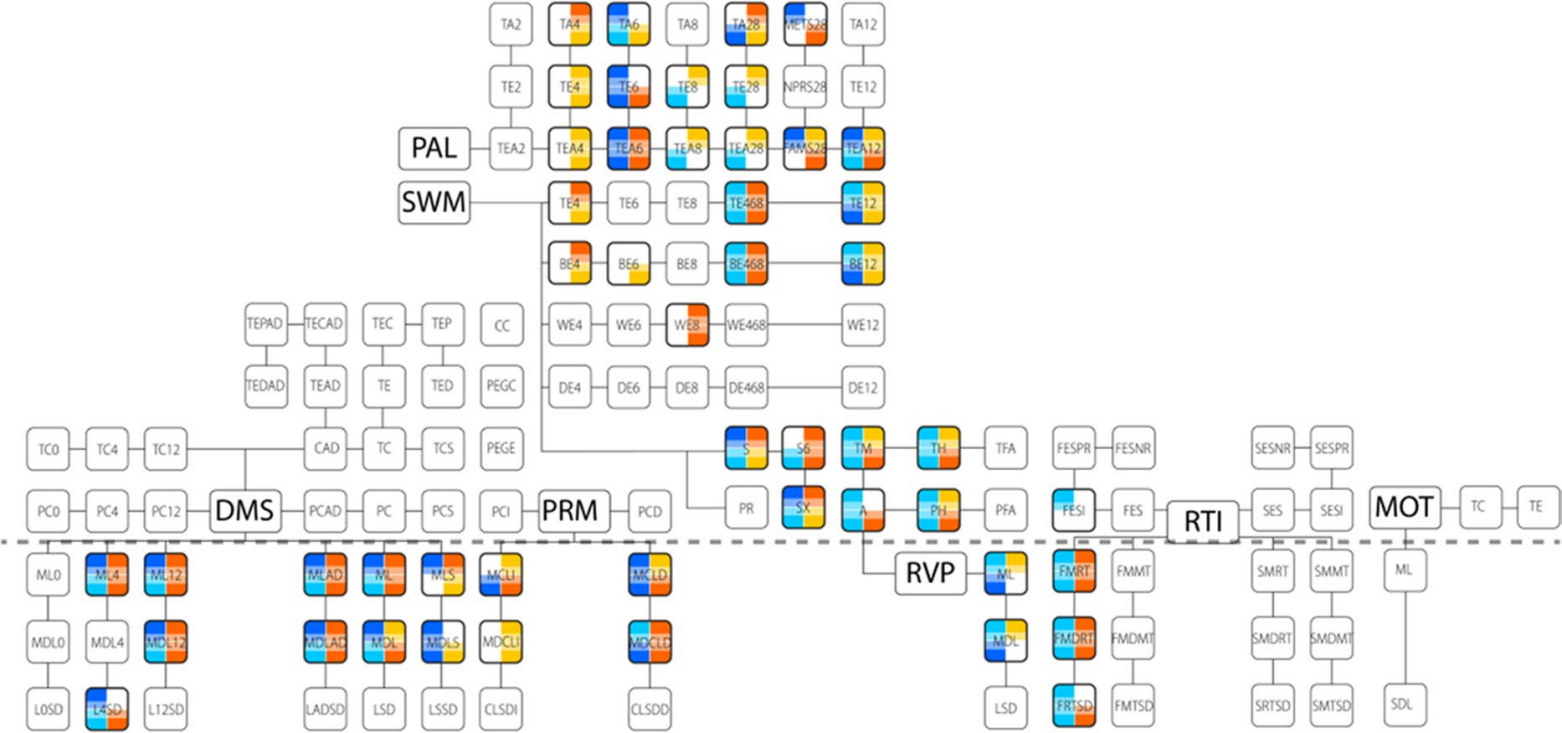
Summary of all CANTAB output variables considered for all 7 tasks. The upper portion, separated by a dashed line, displays accuracy-related indices, while the lower part illustrates response time-like measures. The spatial arrangement of these tasks and their associated measures to one another visually represent variable similarity between each battery, regarding execution and workload. Connecting lines denote similar measures (e.g., median, mean, and standard deviation of response times). Colored squares denote measures where significant associations were found between the measures and connectivity; the top two squares denote the mean, while the bottom two pertain to variance of *DCCC*(*s*). Following the color scheme utilized in previous figures, shades of blue and orange denote the young and elderly group, respectively, with darker shades denoting positive, and lighter shades negative associations

However, distilling general patterns from these outcome measures is not trivial, as some of them are redundant (e.g., mean or median of correct responses), inversely related (e.g., the number of correct responses is complimented by the number of errors), or a positive correlation with connectivity might in fact represent an inverse relationship to performance (e.g., positive correlation between connectivity and response latency implies an inverse relationship, as better performance is characterized by lower RT). In what follows we apply the same grouping of tasks based on their similarity as above, however instead on focusing on topological associations, we discuss most relevant output measures individually for each test and their association to dFrC in general. A summary of the results is presented in Fig. 6, while the full list and details of correlations between CANTAB output variables and dFrC measures (including DFA exponents) is provided in Supplementary Tables S2-S7 in tabular format.

For DMS, response times were measured in various difficulty settings, including simultaneous (denoted by S in the metric registry) and delayed (0-, 4-, or 12-second latency) pattern presentation, both individually and all conditions combined. Across increasing difficulty levels, the mean (ML*) and median (MDL*) response times demonstrated predominantly negative associations with interhemispheric dFrC. Notably, standard deviation of response times (*SD metrics) showed no significant associations, except for the medium difficulty level (4-s delay, L4SD), which displayed similar cortical pattern of associations. In general, RT in more difficult workloads were negatively associated with more elaborate dFrC patterns regarding both *μ*_*DCCC*(*s*)_ and ${\sigma}_{DCCC(s)}^2$. This trend was more pronounced in the elderly group, except for standard deviation, where the observed dFrC patterns of the young group displayed increased positive correlations with the metrics. The PRM and DMS tests might appear somewhat similar in nature; however, the former also tests long-term, while the latter only short-term and working visual memory. Consequently, the two measures might associate with different FrC-patterns. PRM measured less metrics overall and exhibited notably fewer correlations with dFrC compared to DMS regarding both immediate and delayed difficulty levels (denoted by I and D characters, respectively, in the metric register). Similarly, only mean (MCL*) and median (MDCL*) metrics of correct answer RTs were associated with dFrC in both groups. In the less demanding immediate recall variation, both *μ*_*DCCC*(*s*)_ and ${\sigma}_{DCCC(s)}^2$ in the elderly group were mostly negatively associated with RT, while dFrC in the young group showed no correlations, except for ${\sigma}_{DCCC(s)}^2$ with median RT, where fronto-occipital connections were negatively associated with response time. As task difficulty increased, more dFrC patterns associated with performance emerged in both groups, with connectivity strength of most connections becoming negatively correlated with RT (and thus positively with performance).

In the RVP task, mean and median RT (ML and MDL, respectively), total number and percentage of hits (TH, PH), as well as total number of misses (TM) were measured along with a task-specific strategy index (A). Interhemispheric temporal and frontal connections were shown to be negatively associated with accuracy regarding *μ*_*DCCC*(*s*)_ in both groups. Meanwhile, ${\sigma}_{DCCC(s)}^2$ exhibited between-group disparities, wherein the young group was negatively associated with accuracy and response time, but the only significant intertemporal connection was shown to be positively associated with accuracy in the elderly group. The RTI task included two distinct difficulty levels, namely one- (S* metrics) and five-choice (F* metrics) scenarios. Only the more difficult, five-choice task was associated with dFrC patterns in both ACC (FESI) and all recorded RT (FMRT, FMDRT, FMTSD) metrics. As such, the score-specific patterns aligned closely with the unique task-specific topology previously outlined, which highlighted increased frontal dominance compared to the DMS and PRM tasks as well as the increased prevalence of negatively associated connections regarding response time and accuracy in the young group.

In the visuospatial-oriented PAL task, ACC-based metrics (total number of attempts, TA; total number of errors, TE; adjusted number of errors, TEA) were recorded both individually for various difficulties (2, 4, 6, 8, and 12 patterns) and summarized for all difficulty levels except the hardest setting (i.e., from 2 to 8 patterns, TE28, TA28, TEA28, FAMS28, METS28). Similar to the PRM task, the simplest difficulty setting showed no associations with dFrC in either group; however as difficulty increased, associations emerged in the elderly group between TA, TE, and TEA metrics and ${\sigma}_{DCCC(s)}^2$ of frontal and temporal, interhemispheric connections. Further increase of difficulty (4, 6 targets) resulted in associations also manifesting in the young group, with the correlated dFrC patterns becoming more global, with *μ*_*DCCC*(*s*)_ correlating with increased performance in the young and with decreased performance in the elderly groups. On the other hand, ${\sigma}_{DCCC(s)}^2$ was ubiquitously correlated with decreased performance. With substantial increase in difficulty (8 targets), associations became sparser, mostly observed in ${\sigma}_{DCCC(s)}^2$ in the young and *μ*_*DCCC*(*s*)_ regarding in the elderly groups. At the highest difficulty level; however, both *μ*_*DCCC*(*s*)_ and ${\sigma}_{DCCC(s)}^2$ showed more associations with performance compared to the preceding level. Notably, these were overwhelmingly positively associated with the error count in the young group as well, a shift from the negative association observed in lower difficulties. In the summarized metrics, associations between dFrC and performance were sparse, showing positive associations with the number of attempts and—consequently—negative associations with error score. Finally, in SWM also only accuracy-based metrics (within-, between-, delay-, and total-errors, WE, BE, DE, and TE, respectively, along with strategy scores SX, S6, and S) were obtained, individually (for 4, 6, 8, 12 targets) and summarized for all but the most challenging setting. Among these, delay errors exhibited no associations with dFrC at any level. Within-error metrics displayed sparse associations, only in the second hardest difficulty. Between-errors, meanwhile, demonstrated consistent association with dFrC in all but the second hardest difficulty level, mirroring the patterns seen in total errors. Thus, topological changes related to difficulty will be illustrated through this latter metric. In SWM, similar to PAL, only a limited number of associations were observed between and ${\sigma}_{DCCC(s)}^2$ at lower difficulties. Precisely, *μ*_*DCCC*(*s*)_ was found being positively, while ${\sigma}_{DCCC(s)}^2$ being negatively associated with error rates in the elderly group on low difficulty. This pattern reversed at the highest difficulty level, where interhemispheric associations increased; error scores negatively correlated with *μ*_*DCCC*(*s*)_ in both groups, with positive correlations with ${\sigma}_{DCCC(s)}^2$, observed in the young group only. Notably, for summary metrics TE468 and BE468, both *μ*_*DCCC*(*s*)_ and ${\sigma}_{DCCC(s)}^2$ were found negatively associated in the young, while the opposite pattern emerged in the elderly group. Strategy scores showed associations with a global dFrC pattern with a distinct frontal dominance that was especially prevalent in the elderly group. These associations were generally positive with *μ*_*DCCC*(*s*)_ and negative with ${\sigma}_{DCCC(s)}^2$.

## Discussion

It has been shown [3] and confirmed [26] previously that CANTAB measures obtained from the seven administered tasks reveal an age-related decline in cognitive performance even in a healthy population. Csipo and colleagues suggested that impaired peripheral vascular health was a predictor of cognitive decline [3], while in our previous paper, we found that reduced cognitive scores were associated with altered fractal scaling exponent of functional connections. Even though our previous approach established a link between aging, cognitive decline and FrC, that analysis focused on fractal temporal scaling in connectivity, but not connectivity strength itself. In the present work one of our main goals was to alleviate this limitation. As between-group differences in cognitive test scores in the same cohort had already been reported previously [26], here, we omit the detailed discussion of these results; instead, we refer the reader to the original article.

### Dynamic fractal connectivity in healthy aging

Our analyses implied that fractal connectivity strength, on a connection-by-connection basis is quite similar in the two age groups. This result appears to be in contrast with those reporting age-related changes in FC by other studies [59–61]; however, (*i*) fractal connectivity strength has not been investigated in healthy aging previously, as well as (*ii*) our current results could be expected to some extent, as we found no difference previously in cross-spectral power between young and elderly participants in the same cohort [26]. Additionally, it has also been reported that only structural but not functional connectivity might be associated with healthy aging [9]. Nevertheless, a more elaborate machine learning approach—taking all connections into consideration at the same time—indeed revealed a subset of connections that could discriminate between young and elderly individuals with high cross-validation (CV) accuracy. This indicates that not only fractal scaling of connectivity is reduced in aging [26], but also FrC patterns change with age in terms of strength, as well as to some extent, temporal variance. However, before we further discuss these findings, we should address certain specifics of the employed approach to clarify interpretation. Our exploration of the input parameter space indicated that the revealed separability was robust and not specific to regularization parameter *C*, as even though the number of identified connections varied to a larger extent (from 56 to 113, see Table 1), performance was comparable in all cases (in the range of 87 and 90% sample-wise). Notably, outcome measures at *C* = 10 and *C* = 100 were found to match exactly. We implemented our pipeline in Python 3.7.3 using the support vector classification method, abbreviated to SVC, of the Scikit-Learn library with a linear kernel. In this implementation, *C* is in fact the inverse of the constant *λ* of the regularization term in the cost function of an SVM [55]. Therefore, the most likely explanation for this result is that the regularization term was already rendered negligible at *C* = 10 (*λ* = 10^−1^), and thus further increasing *C* had no additional effect on classifier performance. Also, during the SVM-RFE, all time point estimates from all subjects were utilized in order to provide a large enough sample size for the vast number of features. However, the utilized SVM model treated all examples as independent, thus practically disregarding any dependence found in the temporal evolution of FrC patterns. Consequently, the subset of connections identified could discriminate young and elderly individuals based on the mean but not on the variance of *DCCC*(*s*). In fact, connections with stronger temporal fluctuations were penalized as they produce less reliable sample-wise predictions. With these considerations in mind, our analyses regarding ${\sigma}_{DCCC(s)}^2$ should be considered exploratory. This also provides a partial explanation on why variance of only two connections (among the reduced set) were significantly different between the two age groups (Table 2). Nevertheless, ${\sigma}_{DCCC(s)}^2$ still showed strong associations with cognitive functions, underscoring its relevance for better understanding age-related cognitive decline. Therefore, as the presented results indicate the relevance of dFrC in aging [62], more research is called for employing machine learning models [63] able to take into account temporal information for identifying relevant dynamic connections, such as recurrent neural networks or long short-term memory architectures [64–66]. Finally, we need to address another limitation of this approach. Precisely, even though performance was evaluated in a CV setting, the whole dataset was utilized to obtain the feature ranking, which was then evaluated again (also in a CV setting) to obtain the number of connections best separating the two groups. Therefore, our proposed model is not viable for automated classification of young and elderly individuals based on EEG data only, and we would expect a drop in performance if tested for classifying newly obtained recordings as young or elderly. However, we did not utilize SVM-RFE for that purpose in this study, but instead to identify a subset of connections that can best separate the two (or more) populations, such as in [48]. Indeed, for this goal SVM-RFE performed well, providing a subject-wise classification accuracy of 92% on average—comparable with that reported in other studies, e.g., [67]—, in contrast to the marginal difference identified by traditional statistical analysis considering every dFrC measure independently.

The SVM-RFE approach with the best performance identified a set of 56 connections distinguishing young from elderly, although we could not pinpoint a characteristic pattern in terms of temporal scale or cortical location. In general, connections in the frontal cortex mostly showed positive *DCCC*(*s*), while longitudinal connections appeared to be anticorrelated. Connectivity strength (captured as the absolute value of *μ*_*DCCC*(*s*)_) was predominantly higher in the elderly group, implying stronger functional cooperation between brain regions in 41 out of 56 connections. This dominance appeared to be reversed at *s* = 16 and balanced at *s* = 32 (time scales corresponding to 16 and 8 Hz) where instead connection strength was rather higher in the young group for most connections. These results are in partial agreement with those published previously, indicating increased functional connectivity in healthy aging (when compared to healthy young) over a broad range of frequencies and cortical regions [68], while contradictory findings have also been reported [67, 69]. In fact, the majority of former studies reported a decrease instead of an increase of FC in aging [7]. However, most of the utilized approaches did not take into consideration that scaling of fractal dynamics (i.e., long-term autocorrelation) was shown to be reduced in aging [21, 23, 26, 34]. Given that strong autocorrelation can result in spurious estimates of cross-correlation [33], this commonly found age-related weakening in FC might be explained by the concurrent reduction of scale-free dynamics. On the other hand, DCCC normalizes the covariance estimate by the scale free measure of the individual signals (see Eq. (4)) and thus likely eliminates this bias. With this in mind, our results might suggest that reduction of FC in aging might be confounded by the weakened autocorrelation of neural dynamics, as DCCC rather implied higher connectivity strength in the elderly. Nevertheless, this hypothesis warrants further, more elaborate research in the future. Other sources of discrepancy are also most likely related to the different analysis strategies for FC estimation; in our study not only did we compute fractal connectivity, but our evaluation included negative correlations, too, that are most often excluded from analysis [70]. Nevertheless, the observed increase in connectivity strength might reflect a compensatory mechanism [71] for loss in structural connectivity in order to maintain healthy cognition [72].

**Fig. 7:**
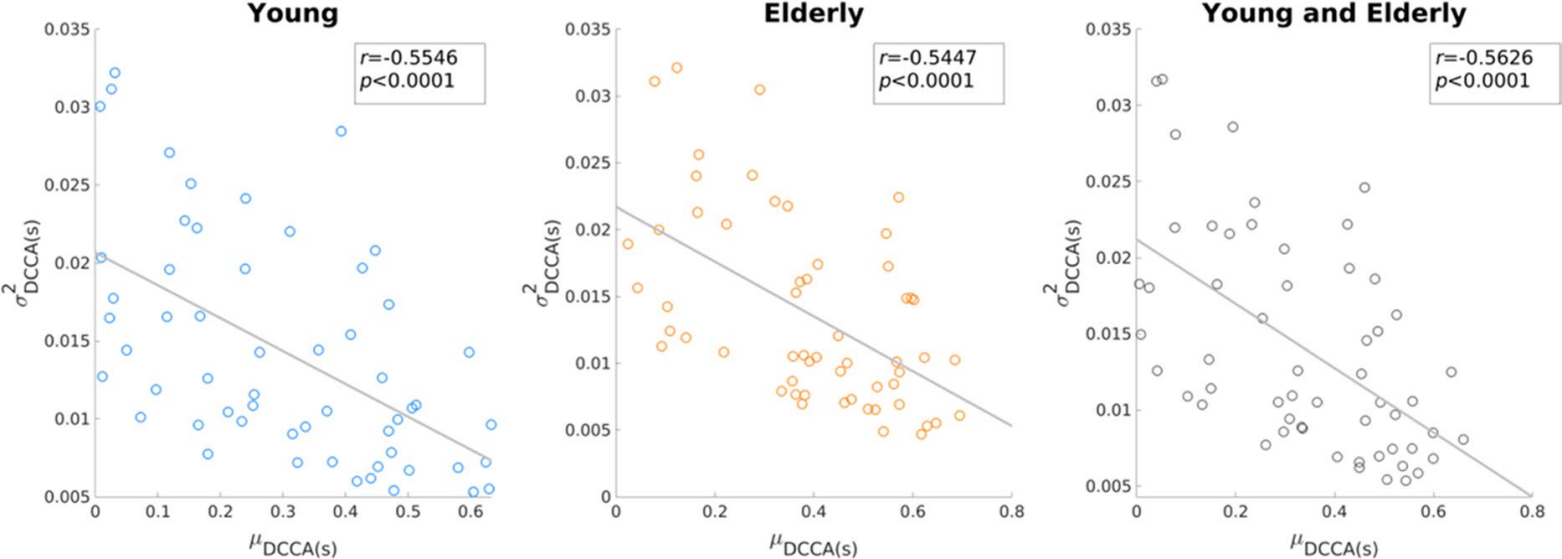
Correlation of ∣*μ*_*DCCC*(*s*)_∣ and ${\sigma}_{DCCC(s)}^2$ in the young (left) and elderly (middle) groups, as well as combining both age groups (right). The solid line illustrates the ordinary least squares regression line

Previous dynamic functional connectivity (DFC) approaches identified reduced temporal variability in aging [30–32]. Our results appear to be in line with these in terms of FrC dynamics and generally reduced ${\sigma}_{DCCC(s)}^2$ values in the elderly group (see Fig. 1.). Notably, ${\sigma}_{DCCC(s)}^2$ and *μ*_*DCCC*(*s*)_ appeared to express opposite patterns in the two groups, with those connections with higher connectivity strength in one group mostly expressing stronger temporal variability in the other (Fig. 1).The variance of *DCCC*(*s*) was decreased in general in the elderly population. Although DFC approaches to healthy aging are yet sparse, similar patterns have been observed before [31]. To understand this inverse relationship between *μ*_*DCCC*(*s*)_ and ${\sigma}_{DCCC(s)}^2$, we computed their Pearson cross-correlation coefficient. Apparently, the mean (as ⟨|*DCCC*(*s*)|⟩) and variance of fractal connectivity was inversely related, with stronger functional couplings being less volatile over time, however this pattern appeared to be comparable (*r* =  − 0.5546 and *r* =  − 0.5447) in the young and elderly groups, respectively (Fig. 7). A similar relationship was found between the long-term autocorrelation and degree of multifractality in delta-band connections [18, 28]. Our present results indicate that this interesting pattern is a characteristic of functional connectivity dynamics that remains unaffected by age; however, better understanding its physiological relevance requires further research.

### Mono- and multifractal dynamics of neural activity

One advantage of utilizing the matrix-notation formula for multi-channel rtDCCA analysis is that DFA-exponents—characterizing fractal temporal scaling in the univariate signals individually [73]—are also obtained simultaneously [40]. Since in this study, utilizing a sliding window approach, we reconstructed the temporal evolution of DFA-exponents, thus allowing for evaluating not only their means but how much they vary over time. In this case—when fractal scaling becomes a local instead of a global property—the process is deemed multifractal [74, 75]. It has been suggested before that mono- and multifractal properties of neural activity change with age and reflect a maturation of the brain at an early age [76], as well as such properties are affected in the case of MCI or AD [29, 77]. Nevertheless, the plausible effect of healthy aging on multifractal characteristics of resting-state EEG has only been addressed scarcely [23].

Regarding the monofractal characteristic of regional neural activity, in our previous study we found reduced fractal scaling exponents over 7 out of 14 brain regions in the elderly when compared to young individuals [26]. As expected, we replicated this outcome in this analysis with a different (time domain) approach; however, in this case mean DFA-exponents were found reduced in 11 out of 14 regions. Similar age-related reduction in spectral slope has been identified by previous studies, too [21, 34]. In our previous study, we utilized multiple-resampling cross-spectral analysis (MRCSA) to obtain cross-spectral scaling exponents characterizing fractal connectivity [78]. MRCSA is a method designed for separating the fractal and oscillatory components of the cross-power spectrum and thus provides an unbiased estimate of the scaling exponent. Our current results imply that even though the MRCSA approach [78] might provide more precise estimates of the scaling exponent, the sliding-window DCCA evaluation was more sensitive in revealing between group differences. Moreover, we identified a total of 35 variables that correlated with the mean DFA exponent (*μ*_*DFA*_) of brain regions in the elderly group, while this number was only 9 in the young group [26]. Notably, in the elderly group 20 out of these were output measures for the RVP task, again in line with our previous results [26]. These different findings might be the result of statistically more robust estimates in our current analysis approach, as in our previous study spectral slopes from 9 epochs were averaged compared to the 137 overlapping windows utilized here. Furthermore, by computing the variance of DFA exponents over time, we obtained a crude assessment on the degree of multifractality (i.e., the spread of the distribution of local scaling exponents [37, 75]) as well. Our results suggest that the degree of multifractality in regional neural activity remains intact in healthy aging; however, more elaborate research would be desirable to confirm this question, addressing it directly with more appropriate tools, such as multifractal DFA [79] or focus-based multifractal formalism [75].

### Fractal connectivity dynamics and cognitive performance

The most important contribution of this work is the identification of associations between fractal connectivity dynamics and cognitive performance in the young and elderly groups. In that regard, here, we extended our previous approach that only considered the fractal scaling exponents of functional connections and regional neural activity [26] and showed that strength and temporal variance of resting-state FrC is also related to cognitive functioning in both young and elderly individuals. In sharp contrast to our previous analysis, where fractal connectivity and neural activity were only found scarcely related to CANTAB scores in the young group—precisely, FrC-CANTAB correlations were significant only in a total of 7 instances [26]—, our current approach indicated widespread associations between dFrC and cognitive performance in both age groups (see Figs. 3, 4, and 5**)**. In fact, significant correlations between CANTAB scores and *μ*_*DCCC*(*S*)_ (all scales combined) were found in 78 and 114 cases in the young and elderly groups, respectively, while in 109 and 130 cases with regards to ${\sigma}_{DCCC(s)}^2$ (a full list of these is reported in the Supplementary Tables S2-S5). Interestingly, despite the fact that connections were primarily selected based on *μ*_*DCCC*(*S*)_ by the SVM-RFE, it appears that the temporal volatility of resting-state FrC shows more and stronger associations to cognitive performance, especially in the elderly group. Despite this, dynamic functional connectivity studies are scarce in the healthy aging population (e.g., [31, 80, 81]), especially those contrasting connectivity dynamics with cognitive performance. In our previous study we demonstrated that long-term memory in functional coupling of distinct brain regions is reduced in healthy aging, and that this reduction correlates with performance in cognitive tasks [26], while here we report that the temporal variance of FrC is also associated with cognitive decline. More specifically, increased variance in frontal connections was mostly found positively, while that of longitudinal connections was found negatively correlated with performance (see Figs. 3, 4, and 5). Taken together, these results emphasize the relevance of not only FC, but connectivity dynamics with regards to age-related cognitive decline, and thus call for more elaborate research in the future.

Figure 6 illustrated the overall pattern of associations between dFrC and all cognitive performance scores in the two age groups, from which we could draw the following conclusions. First, for the DSM, PRM, and RTI tasks only response time measures were found correlated with dFrC measures (except for five-choice error scores in the young group). Second, for a large proportion of the cases, the detected associations were found to be similar in nature in the two groups. Therefore, for those dFrC-CANTAB relations that were identified in both groups, it is reasonable to focus on those that were different in nature (i.e., with opposite sign) in the young and elderly cohorts. Notably, this was the case for ${\sigma}_{DCCC(s)}^2$ and all relevant measures of DMS and RTI, where consistently a lower variance in the young, but higher variance in the elderly group correlated with better performance. This indicates that at a younger age, a more consistent connectivity is ideal for better performance (RT); however, in aging more flexibility in connectivity promotes faster task solving. These findings further support the role of connectivity dynamics in healthy aging and cognitive functioning, an association that has not been shown previously to the best of our knowledge. Third, we could identify those associations that were only characteristic to one age group. Most apparently, performance in the lower difficulty settings of the PAL and SWM tasks (especially with 4-choice problems) correlated with dFrC only in the elderly group (regarding both *μ*_*DCCC*(*s*)_ and ${\sigma}_{DCCC(s)}^2$), while on higher difficulty levels this distinction vanishes, and instead connectivity was similarly related to CANTAB measures in both groups. These results indicate an already diminished cognitive reserve in the elderly population [82], showing that a smaller mental workload already poses a significant challenge reflected in fractal connectivity dynamics that is yet absent in the young group.

### Limitations and future perspectives

Our analyses revealed an elaborate pattern of dFrC-CANTAB score associations. However, we should refrain from drawing conclusions on how connectivity between specific cortical regions drives various cognitive domains in healthy aging, given the limited spatial resolution of our experimental setup (14-channel EEG). Furthermore, even though we found frontal connections to be more prevalently related to cognitive performance, this observation is most likely biased by the fact that electrode density was also higher over those regions, naturally providing more details compared to, e.g., motor, parietal and occipital cortices. The same argument holds for the topology that was revealed by the subset of connections most discriminative between young and elderly populations. Therefore, a better understanding of the phenomena addressed here could only be achieved by confirming our results with higher spatial resolution, where source reconstruction is also applicable [83]. Furthermore, our analysis of correlations between dFrC measures and CANTAB output variables should be considered exploratory, as our intention was to cover a wide range of cognitive aspects that might be affected in healthy aging, instead of focusing on a few selected measures. However, our results—complementing those reported in [26]—provide relevant guidance for future studies on which cognitive functions to put more emphasis on. Namely, response time is ubiquitously affected in healthy aging but appears as a compensatory mechanism [71] for reduced processing speed in order to maintain precise task solving [84, 85], as well as performance is already challenged even regarding lower difficulty settings in the elderly population (e.g., [86]). Another aspect we did not address was the possible confounding effect of education on the outcomes. Volunteers for both groups were recruited at Semmelweis University; thus, most members of the elderly group possessed a university degree (or higher), while the young group was mainly composed of undergraduate and graduate students. Therefore, even though both groups could be considered well-educated, differences in education would be also strongly correlated with group assignment, simply reflecting that most individuals in the young group could not obtain a university degree yet due to their age. We believe these effects to be negligible in our study; however, this aspect should also be more rigorously assessed and controlled for in future studies. It is also important to highlight that even though our study is one of the first to report fractal connectivity alterations in healthy aging, we did not contrast our results with those obtained with another, ‘conventional’ FC estimator (see [87] for a review). Given the extensive nature of the utilized analysis pipeline, we concluded that introducing another FC estimator would overburden the study and thus it is beyond its scope. Nevertheless, comparing FrC measures with those obtained by conventional FC analyses is a relevant research question – especially in light of the somewhat contradictory nature of our present findings – and warrants further investigation. Finally, identifying the neurophysiological correlates of age-related cognitive decline is not only important for better understanding its pathophysiology, but also to identify potential targets and develop preventive/therapeutic intervention strategies, which we intend to focus on in future research.

## Conclusions

In this study, we analyzed dynamic fractal connectivity in young and healthy elderly individuals. Utilizing a machine learning-based feature selection method, we identified a subset of connections best discriminating between the two age groups, yielding much better performance than considering connections individually. Our analysis of how fractal connectivity dynamics in this subset are related to cognitive performance in a wide range of domains revealed key insights. In most tasks, lengthened response latency was associated with connectivity in both groups, as well as changes in dFrC reflected reduced cognitive performance in the elderly group even on lighter difficulty settings. Interestingly, the temporal variance of FrC revealed more distinctive associations with performance, as the identified correlations were different in nature in the two age groups. Despite its limitations, our results—in accordance with those reported in [26]—underscore the importance of fractal connectivity and a dynamic connectivity approach in understanding aging-related cognitive decline, and thus, they call for future research targeting this issue in a more elaborate setting.

### Supplementary information

ESM 1 (DOCX 82 kb)
