# Supplementary material for: Fingerprints of decreased cognitive performance on fractal connectivity dynamics in healthy aging
Source: GeroScience. 2023 Dec 20;46(1):713–36. doi: 10.1007/s11357-023-01022-x (PMC10828149; doi:10.1007/s11357-023-01022-x)
Supplement: Supplementary file 1 — (DOCX 82 kb) [file 11357_2023_1022_MOESM1_ESM.docx]

**Supplementary Table S1.** All significant between-group differences in CANTAB test scores.

| CANTAB | E_young | E_elderly | test | *p*-value |
| --- | --- | --- | --- | --- |
| DMSL4SD | 827.6409 | 1456.715 | ranksum | 0.042663 |
| DMSMDL | 2284 | 3459 | ranksum | 0.012187 |
| DMSMDL12 | 2513.25 | 3860 | ranksum | 0.009675 |
| DMSMDLAD | 2287.75 | 3586 | ranksum | 0.024058 |
| DMSMDLS | 2252.5 | 3238.868 | ranksum | 0.001031 |
| DMSML | 2820.375 | 3677.531 | ranksum | 0.02705 |
| DMSML12 | 2846.1 | 4322 | ranksum | 0.018941 |
| DMSML4 | 2475.667 | 3634.2 | ranksum | 0.021848 |
| DMSMLAD | 2487.033 | 3956.667 | ranksum | 0.018384 |
| DMSMLS | 2403.723 | 3276.616 | ranksum | 0.003333 |
| PALFAMS28 | 16.54167 | 11.94737 | ranksum | 0.000405 |
| PALFAMS28Percentile | 66.5 | 44.05263 | ranksum | 0.019712 |
| PALFAMS28ZScore | 0.624167 | -0.16842 | ranksum | 0.01775 |
| PALMETS28 | 1 | 2 | ranksum | 0.001269 |
| PALTA28 | 6 | 8 | ranksum | 0.003231 |
| PALTA4 | 1 | 1 | ranksum | 0.024143 |
| PALTA6 | 1 | 2 | ranksum | 0.00134 |
| PALTE28 | 5 | 13.15789 | ranksum | 0.001412 |
| PALTE4 | 0 | 0 | ranksum | 0.025123 |
| PALTE6 | 0 | 3 | ranksum | 0.001238 |
| PALTE8 | 4.041667 | 8.157895 | ranksum | 0.031411 |
| PALTEA12 | 5.5 | 12 | ranksum | 0.018524 |
| PALTEA28 | 5 | 13.15789 | ranksum | 0.001271 |
| PALTEA4 | 0 | 0 | ranksum | 0.024539 |
| PALTEA6 | 0 | 3 | ranksum | 0.000991 |
| PALTEA8 | 4.041667 | 8.157895 | ranksum | 0.030782 |
| PRMMCLD | 1667.148 | 2082.149 | ranksum | 0.012308 |
| PRMMCLI | 1516.364 | 1900.687 | ranksum | 0.014247 |
| PRMMDCLD | 1531 | 1927.842 | ranksum | 0.00329 |
| PRMMDCLI | 1280.25 | 1744 | ranksum | 0.001483 |
| RTIFESI | 0 | 0 | ranksum | 0.024359 |
| RTIFMDRT | 334.3542 | 383.7895 | ranksum | 0.00225 |
| RTIFMRT | 341.5054 | 391.4468 | ranksum | 0.002171 |
| RTIFRTSD | 36.54223 | 46.89996 | ranksum | 0.02276 |
| RVPA | 0.9398 | 0.8971 | ranksum | 0.014329 |
| RVPAPercentile | 59.95833 | 36.26316 | ranksum | 0.012574 |
| RVPAZScore | 0.345417 | -0.42421 | ranksum | 0.01861 |
| RVPMDL | 411.5 | 528 | ranksum | 0.000164 |
| RVPML | 466.6207 | 568 | ranksum | 0.001015 |
| RVPPH | 0.743063 | 0.614037 | ranksum | 0.042279 |
| RVPTH | 40.125 | 33.15789 | ranksum | 0.04149 |
| RVPTM | 13.875 | 20.84211 | ranksum | 0.040707 |
| SWMBE12 | 14 | 35 | ranksum | 0.008249 |
| SWMBE4 | 0 | 2 | ranksum | 0.019197 |
| SWMBE468 | 5 | 19 | ranksum | 0.018119 |
| SWMBE6 | 0 | 6 | ranksum | 0.027662 |
| SWMS | 6.708333 | 8.947368 | ranksum | 0.03024 |
| SWMS6 | 3 | 4 | ranksum | 0.013901 |
| SWMSX | 11.375 | 15.52632 | ranksum | 0.024136 |
| SWMTE12 | 14 | 35 | ranksum | 0.011275 |
| SWMTE4 | 0 | 2 | ranksum | 0.018557 |
| SWMTE468 | 6.375 | 15.78947 | ranksum | 0.008568 |
| SWMTE6 | 0 | 6 | ranksum | 0.031193 |
| SWMWE8 | 0 | 0 | ranksum | 0.028357 |

**Supplementary Table S2.** All significant correlations between $\mu_{DCCC\left( S \right)}$ and CANTAB scores in the young group.

| CANTAB | ch*1* | ch*2* | scale | E_cantab | E_dccc | *p*-value | *r* |
| --- | --- | --- | --- | --- | --- | --- | --- |
| PALTA6 | F3 | P8 | 16 | 1 | -0.45181 | 0.000808 | 0.63737 |
| PALTA6 | F3 | P8 | 64 | 1 | -0.35682 | 0.001171 | 0.622122 |
| PALTE6 | F3 | P8 | 16 | 0 | -0.45181 | 0.001479 | 0.61208 |
| PALTEA6 | F3 | P8 | 16 | 0 | -0.45181 | 0.001479 | 0.61208 |
| PALTE6 | F3 | P8 | 64 | 0 | -0.35682 | 0.002789 | 0.583028 |
| PALTEA6 | F3 | P8 | 64 | 0 | -0.35682 | 0.002789 | 0.583028 |
| PALTA6 | F3 | P8 | 8 | 1 | -0.41828 | 0.003005 | 0.579427 |
| PALTA6 | F3 | P8 | 128 | 1 | -0.311 | 0.003133 | 0.577394 |
| RVPML | FC5 | F8 | 64 | 466.6207 | -0.14262 | 0.004068 | 0.572174 |
| RTIFMRT | T8 | FC6 | 64 | 341.5054 | 0.240095 | 0.004483 | -0.56696 |
| DMSL4SD | F3 | T7 | 16 | 827.6409 | 0.011412 | 0.005172 | -0.55913 |
| PALTE6 | F3 | P8 | 8 | 0 | -0.41828 | 0.005467 | 0.548968 |
| PALTEA6 | F3 | P8 | 8 | 0 | -0.41828 | 0.005467 | 0.548968 |
| PALTE6 | F3 | P8 | 128 | 0 | -0.311 | 0.007137 | 0.534443 |
| PALTEA6 | F3 | P8 | 128 | 0 | -0.311 | 0.007137 | 0.534443 |
| PALTEA12 | O2 | T8 | 8 | 5.5 | 0.315089 | 0.007374 | -0.53262 |
| RVPMDL | FC5 | F8 | 64 | 411.5 | -0.14262 | 0.008888 | 0.527826 |
| DMSML4 | F3 | T7 | 16 | 2475.667 | 0.011412 | 0.010105 | -0.52 |
| DMSMDLS | AF3 | F4 | 16 | 2252.5 | 0.370319 | 0.012116 | -0.50359 |
| RVPML | P8 | FC6 | 64 | 466.6207 | 0.022707 | 0.013493 | -0.50174 |
| PALTA6 | FC6 | F8 | 8 | 1 | 0.378896 | 0.016587 | -0.48387 |
| RTIFMRT | T8 | FC6 | 16 | 341.5054 | 0.253468 | 0.017323 | -0.48522 |
| RTIFESI | T7 | T8 | 32 | 0 | -0.2393 | 0.019742 | -0.47245 |
| RVPMDL | P7 | O1 | 128 | 411.5 | 0.597481 | 0.019923 | 0.475652 |
| RTIFMDRT | T8 | FC6 | 64 | 334.3542 | 0.240095 | 0.019959 | -0.47171 |
| DMSML4 | T7 | T8 | 16 | 2475.667 | -0.26271 | 0.020174 | 0.474783 |
| PALTEA12 | O1 | FC6 | 128 | 5.5 | -0.44757 | 0.02128 | 0.467406 |
| RTIFRTSD | F7 | AF4 | 64 | 36.54223 | 0.009805 | 0.021471 | 0.470435 |
| RTIFRTSD | F7 | AF4 | 128 | 36.54223 | -0.02581 | 0.021738 | 0.469565 |
| DMSMLS | AF3 | F4 | 16 | 2403.723 | 0.370319 | 0.022556 | -0.46696 |
| PALTE6 | FC6 | F8 | 8 | 0 | 0.378896 | 0.022598 | -0.46332 |
| PALTEA6 | FC6 | F8 | 8 | 0 | 0.378896 | 0.022598 | -0.46332 |
| SWMS | T7 | FC6 | 8 | 6.708333 | -0.32297 | 0.023435 | 0.46082 |
| SWMTE12 | F3 | O2 | 32 | 14 | -0.45864 | 0.023544 | 0.460501 |
| SWMSX | T7 | FC6 | 8 | 11.375 | -0.32297 | 0.024216 | 0.458554 |
| RTIFMRT | F3 | O2 | 16 | 341.5054 | -0.46887 | 0.025164 | 0.45913 |
| DMSMDL12 | AF3 | F4 | 16 | 2513.25 | 0.370319 | 0.025164 | -0.45913 |
| DMSMDL | AF3 | F4 | 16 | 2284 | 0.370319 | 0.028345 | -0.45043 |
| RTIFESI | T7 | T8 | 64 | 0 | -0.16225 | 0.028565 | -0.44691 |
| RTIFMRT | AF3 | F3 | 64 | 341.5054 | 0.408188 | 0.02868 | -0.44957 |
| SWMTE12 | F3 | O2 | 16 | 14 | -0.46887 | 0.028874 | 0.446137 |
| SWMBE12 | F3 | O2 | 32 | 14 | -0.45864 | 0.02982 | 0.443816 |
| PRMMDCLD | P7 | O1 | 128 | 1531 | 0.597481 | 0.030405 | 0.445217 |
| DMSML4 | T7 | T8 | 32 | 2475.667 | -0.2393 | 0.030759 | 0.444348 |
| RVPAPercentile | T7 | T8 | 64 | 59.95833 | -0.16225 | 0.030974 | -0.44106 |
| RVPAZScore | T7 | T8 | 64 | 0.345417 | -0.16225 | 0.030974 | -0.44106 |
| RTIFRTSD | F7 | AF4 | 32 | 36.54223 | 0.114083 | 0.031479 | 0.442609 |
| RTIFMDRT | F3 | O2 | 16 | 334.3542 | -0.46887 | 0.032978 | 0.436467 |
| SWMBE12 | F3 | O2 | 16 | 14 | -0.46887 | 0.033789 | 0.43467 |
| RTIFESI | FC6 | F4 | 64 | 0 | 0.167137 | 0.03403 | 0.434141 |
| RTIFESI | FC6 | F4 | 128 | 0 | 0.152463 | 0.03403 | 0.434141 |
| RVPPH | T7 | T8 | 64 | 0.743063 | -0.16225 | 0.037009 | -0.42786 |
| RVPTH | T7 | T8 | 64 | 40.125 | -0.16225 | 0.037009 | -0.42786 |
| RVPTM | T7 | T8 | 64 | 13.875 | -0.16225 | 0.037009 | 0.427857 |
| DMSML12 | AF3 | F4 | 16 | 2846.1 | 0.370319 | 0.038162 | -0.42783 |
| DMSMDLAD | F3 | T7 | 16 | 2287.75 | 0.011412 | 0.038588 | -0.42696 |
| SWMBE468 | T7 | T8 | 8 | 5 | -0.2517 | 0.039099 | 0.423684 |
| RTIFESI | T7 | AF4 | 8 | 0 | -0.3359 | 0.040296 | 0.421372 |
| DMSMLAD | T7 | T8 | 16 | 2487.033 | -0.26271 | 0.040331 | 0.423478 |
| DMSML | F3 | T7 | 16 | 2820.375 | 0.011412 | 0.040331 | -0.42348 |
| DMSML | AF3 | F4 | 16 | 2820.375 | 0.370319 | 0.040331 | -0.42348 |
| RVPA | T7 | T8 | 64 | 0.9398 | -0.16225 | 0.040463 | -0.42105 |
| PALMETS28 | FC6 | F8 | 8 | 1 | 0.378896 | 0.040959 | -0.42011 |
| DMSMLAD | AF3 | F4 | 16 | 2487.033 | 0.370319 | 0.041225 | -0.42174 |
| RTIFMRT | T8 | FC6 | 8 | 341.5054 | 0.233974 | 0.042135 | -0.42 |
| PRMMCLD | AF3 | O2 | 128 | 1667.148 | -0.46905 | 0.042596 | -0.41913 |
| DMSMDL | F3 | T7 | 16 | 2284 | 0.011412 | 0.042596 | -0.41913 |
| PALTA6 | AF3 | P8 | 128 | 1 | -0.42705 | 0.043319 | 0.415764 |
| DMSL4SD | F7 | FC5 | 32 | 827.6409 | 0.624282 | 0.043531 | -0.41739 |
| DMSMLAD | F3 | T7 | 16 | 2487.033 | 0.011412 | 0.044481 | -0.41565 |
| RTIFMRT | AF3 | F4 | 16 | 341.5054 | 0.370319 | 0.044963 | -0.41478 |
| RTIFMDRT | AF3 | F4 | 16 | 334.3542 | 0.370319 | 0.045642 | -0.41166 |
| SWMTE468 | T7 | T8 | 8 | 6.375 | -0.2517 | 0.045832 | 0.411333 |
| RTIFMDRT | AF3 | F3 | 64 | 334.3542 | 0.408188 | 0.045894 | -0.41123 |
| PRMMCLD | AF3 | O2 | 64 | 1667.148 | -0.50612 | 0.046432 | -0.41217 |
| DMSMDLAD | T7 | T8 | 16 | 2287.75 | -0.26271 | 0.046432 | 0.412174 |
| RTIFESI | T7 | T8 | 16 | 0 | -0.26271 | 0.047436 | -0.4086 |
| DMSML | F3 | F4 | 8 | 2820.375 | 0.440425 | 0.048966 | -0.40783 |

**Supplementary Table S3.** All significant correlations between $\mu_{DCCC\left( S \right)}$ and CANTAB scores in the elderly group.

| CANTAB | ch_1_ | ch_2_ | scale | E_cantab | E_dccc | *p*-value | *r* |
| --- | --- | --- | --- | --- | --- | --- | --- |
| DMSML4 | AF3 | F4 | 16 | 3634.2 | 0.35769 | 0.0005 | -0.73509 |
| RTIFMDRT | F7 | AF4 | 64 | 383.7895 | 0.162185 | 0.000853 | -0.69974 |
| DMSML | AF3 | F4 | 16 | 3677.531 | 0.35769 | 0.001328 | -0.69474 |
| RTIFMDRT | F7 | AF4 | 128 | 383.7895 | 0.123114 | 0.002134 | -0.65935 |
| DMSMDLAD | AF3 | F4 | 16 | 3586 | 0.35769 | 0.002464 | -0.66491 |
| SWMS | F7 | O2 | 16 | 8.947368 | -0.5281 | 0.003271 | 0.638306 |
| SWMS6 | AF3 | O2 | 128 | 4 | -0.59654 | 0.003292 | 0.637985 |
| DMSML4 | FC5 | F8 | 64 | 3634.2 | -0.16465 | 0.003328 | -0.64912 |
| DMSMLAD | AF3 | F4 | 16 | 3956.667 | 0.35769 | 0.003551 | -0.64561 |
| PALTEA12 | T7 | T8 | 64 | 12 | -0.32122 | 0.003707 | 0.631845 |
| SWMSX | F7 | O2 | 16 | 15.52632 | -0.5281 | 0.004097 | 0.626573 |
| SWMS6 | AF3 | O2 | 64 | 4 | -0.62309 | 0.004732 | 0.618818 |
| SWMS | F7 | AF4 | 32 | 8.947368 | 0.223283 | 0.005134 | -0.61434 |
| SWMS6 | F7 | O2 | 16 | 4 | -0.5281 | 0.00549 | 0.610604 |
| DMSML12 | F4 | AF4 | 128 | 4322 | 0.546312 | 0.006332 | -0.61228 |
| PRMMDCLI | AF3 | O2 | 64 | 1744 | -0.62309 | 0.007097 | 0.595875 |
| SWMWE8 | AF3 | F3 | 8 | 0 | 0.62857 | 0.007119 | -0.59569 |
| DMSMDL | AF3 | F4 | 16 | 3459 | 0.35769 | 0.007303 | -0.60351 |
| PALFAMS28Percentile | O1 | P8 | 8 | 44.05263 | 0.217563 | 0.007577 | -0.59201 |
| PALFAMS28ZScore | O1 | P8 | 8 | -0.16842 | 0.217563 | 0.007577 | -0.59201 |
| RTIFMDRT | F7 | AF4 | 32 | 383.7895 | 0.223283 | 0.009058 | -0.58121 |
| SWMWE8 | F3 | O2 | 16 | 0 | -0.45372 | 0.009176 | -0.58041 |
| RTIFMRT | FC5 | AF4 | 128 | 391.4468 | 0.078088 | 0.009354 | -0.58772 |
| DMSML12 | FC6 | F4 | 16 | 4322 | 0.379557 | 0.009354 | -0.58772 |
| RTIFMRT | F7 | AF4 | 64 | 391.4468 | 0.162185 | 0.009868 | -0.58421 |
| PRMMCLI | AF3 | O2 | 64 | 1900.687 | -0.62309 | 0.009868 | 0.584211 |
| SWMTE12 | T7 | T8 | 32 | 35 | -0.38571 | 0.010912 | 0.569548 |
| DMSMDL12 | T7 | FC6 | 8 | 3860 | -0.50979 | 0.010966 | 0.577193 |
| SWMSX | F7 | AF4 | 32 | 15.52632 | 0.223283 | 0.011782 | -0.56462 |
| SWMBE4 | T7 | FC6 | 8 | 2 | -0.50979 | 0.011784 | -0.56461 |
| SWMTE4 | T7 | FC6 | 8 | 2 | -0.50979 | 0.011784 | -0.56461 |
| DMSMLAD | FC5 | F8 | 64 | 3956.667 | -0.16465 | 0.013124 | -0.56491 |
| DMSML12 | FC6 | F4 | 64 | 4322 | 0.364076 | 0.0138 | -0.5614 |
| SWMWE8 | F3 | O2 | 32 | 0 | -0.44912 | 0.014289 | -0.5519 |
| PALTE8 | O1 | P8 | 8 | 8.157895 | 0.217563 | 0.014344 | 0.551643 |
| PALTEA8 | O1 | P8 | 8 | 8.157895 | 0.217563 | 0.014344 | 0.551643 |
| DMSML | FC5 | F8 | 64 | 3677.531 | -0.16465 | 0.014504 | -0.55789 |
| DMSML | AF3 | O2 | 64 | 3677.531 | -0.62309 | 0.016 | 0.550877 |
| DMSMDL12 | F4 | AF4 | 128 | 3860 | 0.546312 | 0.017201 | -0.54561 |
| PRMMDCLI | AF3 | O2 | 128 | 1744 | -0.59654 | 0.01751 | 0.537955 |
| SWMBE12 | T7 | T8 | 32 | 35 | -0.38571 | 0.018284 | 0.534915 |
| DMSMDL | AF3 | O2 | 64 | 3459 | -0.62309 | 0.018914 | 0.538596 |
| PALFAMS28 | O1 | P8 | 8 | 11.94737 | 0.217563 | 0.02 | -0.52852 |
| RTIFMRT | F7 | AF4 | 128 | 391.4468 | 0.123114 | 0.020287 | -0.53333 |
| RTIFMDRT | FC5 | AF4 | 128 | 383.7895 | 0.078088 | 0.020737 | -0.5259 |
| PALTEA12 | T7 | T8 | 16 | 12 | -0.40512 | 0.021365 | 0.523731 |
| PALTE8 | O1 | P8 | 64 | 8.157895 | 0.275489 | 0.023063 | 0.518103 |
| PALTEA8 | O1 | P8 | 64 | 8.157895 | 0.275489 | 0.023063 | 0.518103 |
| DMSML12 | FC6 | F4 | 8 | 4322 | 0.363463 | 0.023274 | -0.52281 |
| DMSML4 | F3 | F4 | 8 | 3634.2 | 0.461955 | 0.023804 | -0.52105 |
| SWMS6 | P8 | FC6 | 64 | 4 | -0.0864 | 0.023826 | -0.51568 |
| PRMMCLI | AF3 | O2 | 128 | 1900.687 | -0.59654 | 0.024344 | 0.519298 |
| DMSMLAD | F3 | T7 | 128 | 3956.667 | -0.16712 | 0.025453 | 0.515789 |
| PALTEA12 | T7 | T8 | 32 | 12 | -0.38571 | 0.026454 | 0.50778 |
| SWMTE12 | T7 | T8 | 64 | 35 | -0.32122 | 0.026948 | 0.506362 |
| RTIFMDRT | FC5 | AF4 | 8 | 383.7895 | 0.14124 | 0.02718 | -0.50571 |
| SWMS6 | F7 | AF4 | 32 | 4 | 0.223283 | 0.027855 | -0.50382 |
| SWMWE8 | AF3 | F3 | 64 | 0 | 0.587555 | 0.028514 | -0.50201 |
| SWMSX | AF3 | O2 | 128 | 15.52632 | -0.59654 | 0.029584 | 0.499135 |
| RTIFMRT | FC5 | AF4 | 8 | 391.4468 | 0.14124 | 0.029656 | -0.50351 |
| DMSMDL | AF3 | F3 | 8 | 3459 | 0.62857 | 0.030298 | -0.50175 |
| PALFAMS28Percentile | O1 | P8 | 64 | 44.05263 | 0.275489 | 0.030683 | -0.49627 |
| PALFAMS28ZScore | O1 | P8 | 64 | -0.16842 | 0.275489 | 0.030683 | -0.49627 |
| PRMMCLD | FC6 | F4 | 64 | 2082.149 | 0.364076 | 0.031617 | -0.49825 |
| SWMBE468 | T7 | FC6 | 8 | 19 | -0.50979 | 0.032704 | -0.4912 |
| SWMBE12 | T7 | T8 | 64 | 35 | -0.32122 | 0.032787 | 0.490997 |
| RVPMDL | T7 | T8 | 64 | 528 | -0.32122 | 0.032981 | 0.494737 |
| SWMTE468 | T7 | FC6 | 8 | 15.78947 | -0.50979 | 0.033154 | -0.4901 |
| SWMS | AF3 | O2 | 128 | 8.947368 | -0.59654 | 0.033177 | 0.490049 |
| RVPAPercentile | FC5 | O1 | 8 | 36.26316 | -0.09205 | 0.033602 | 0.489026 |
| RVPAZScore | FC5 | O1 | 8 | -0.42421 | -0.09205 | 0.033602 | 0.489026 |
| PRMMCLD | FC5 | AF4 | 128 | 2082.149 | 0.078088 | 0.033681 | -0.49298 |
| PRMMCLD | T8 | FC6 | 16 | 2082.149 | 0.391079 | 0.033681 | 0.492982 |
| PALTA4 | F3 | F4 | 8 | 1 | 0.461955 | 0.033835 | 0.488468 |
| SWMS | AF3 | O2 | 64 | 8.947368 | -0.62309 | 0.034292 | 0.487385 |
| SWMSX | AF3 | P8 | 128 | 15.52632 | -0.55052 | 0.035712 | 0.48409 |
| DMSMDL | AF3 | O2 | 128 | 3459 | -0.59654 | 0.035849 | 0.487719 |
| RTIFMRT | F7 | AF4 | 32 | 391.4468 | 0.223283 | 0.036596 | -0.48596 |
| DMSML | AF3 | O2 | 128 | 3677.531 | -0.59654 | 0.036596 | 0.485965 |
| DMSML12 | FC6 | F4 | 128 | 4322 | 0.346756 | 0.037354 | -0.48421 |
| DMSMDLAD | FC5 | F8 | 64 | 3586 | -0.16465 | 0.037354 | -0.48421 |
| PALTE4 | F3 | F4 | 8 | 0 | 0.461955 | 0.037768 | 0.479491 |
| PALTEA4 | F3 | F4 | 8 | 0 | 0.461955 | 0.037768 | 0.479491 |
| SWMSX | AF3 | O2 | 64 | 15.52632 | -0.62309 | 0.038094 | 0.47878 |
| PRMMCLD | T8 | FC6 | 8 | 2082.149 | 0.376151 | 0.038126 | 0.482456 |
| PALTE28 | O1 | P8 | 8 | 13.15789 | 0.217563 | 0.038848 | 0.477154 |
| PALTEA28 | O1 | P8 | 8 | 13.15789 | 0.217563 | 0.038848 | 0.477154 |
| DMSMLS | AF3 | F3 | 64 | 3276.616 | 0.587555 | 0.038909 | -0.4807 |
| DMSMLS | AF3 | F3 | 8 | 3276.616 | 0.62857 | 0.038909 | -0.4807 |
| DMSMDL | AF3 | F3 | 64 | 3459 | 0.587555 | 0.038909 | -0.4807 |
| SWMWE8 | FC6 | F4 | 16 | 0 | 0.379557 | 0.039131 | 0.47655 |
| PRMMDCLI | F7 | O2 | 16 | 1744 | -0.5281 | 0.039142 | 0.476525 |
| DMSMDL12 | FC6 | F8 | 8 | 3860 | 0.540696 | 0.039706 | -0.47895 |
| PALTEA12 | T7 | T8 | 8 | 12 | -0.38156 | 0.039868 | 0.474991 |
| RVPPH | AF3 | F4 | 16 | 0.614037 | 0.35769 | 0.040537 | -0.47359 |
| RVPTH | AF3 | F4 | 16 | 33.15789 | 0.35769 | 0.040537 | -0.47359 |
| RVPTM | AF3 | F4 | 16 | 20.84211 | 0.35769 | 0.040537 | 0.473594 |
| SWMWE8 | FC6 | F4 | 8 | 0 | 0.363463 | 0.042082 | 0.47044 |
| RVPML | FC6 | F4 | 128 | 568 | 0.346756 | 0.042171 | 0.473684 |
| PRMMCLD | AF3 | O2 | 64 | 2082.149 | -0.62309 | 0.042171 | 0.473684 |
| SWMTE12 | T7 | T8 | 16 | 35 | -0.40512 | 0.04299 | 0.468627 |
| SWMTE12 | T7 | T8 | 8 | 35 | -0.38156 | 0.043434 | 0.467749 |
| SWMBE12 | AF3 | P8 | 128 | 35 | -0.55052 | 0.043672 | 0.467282 |
| DMSML | F7 | AF4 | 32 | 3677.531 | 0.223283 | 0.043881 | -0.47018 |
| RTIFMRT | P8 | T8 | 32 | 391.4468 | 0.573173 | 0.044756 | 0.468421 |
| PRMMDCLD | FC6 | F4 | 64 | 1927.842 | 0.364076 | 0.044756 | -0.46842 |
| DMSMDL12 | FC6 | F4 | 16 | 3860 | 0.379557 | 0.045644 | -0.46667 |
| PALTEA12 | AF3 | F3 | 64 | 12 | 0.587555 | 0.045653 | -0.46347 |
| DMSMDLAD | AF3 | O2 | 64 | 3586 | -0.62309 | 0.047462 | 0.463158 |
| DMSMDL | AF3 | P8 | 128 | 3459 | -0.55052 | 0.048392 | 0.461404 |
| PALTA28 | O1 | P8 | 8 | 8 | 0.217563 | 0.049669 | 0.456115 |
| RTIFMDRT | F7 | O2 | 16 | 383.7895 | -0.5281 | 0.049925 | 0.455663 |
| RVPAPercentile | FC5 | O1 | 16 | 36.26316 | -0.10365 | 0.049925 | 0.455663 |
| RVPAZScore | FC5 | O1 | 16 | -0.42421 | -0.10365 | 0.049925 | 0.455663 |

**Supplementary Table S4.** All significant correlations between $\sigma_{DCCC\left( s \right)}^{2}$ and CANTAB scores in the young group.

| CANTAB | ch_1_ | ch_2_ | scale | E_cantab | E_dccc | *p*-value | *r* |
| --- | --- | --- | --- | --- | --- | --- | --- |
| PALTE6 | FC5 | O1 | 16 | 0 | 0.012606 | 0.001027 | -0.62761 |
| PALTEA6 | FC5 | O1 | 16 | 0 | 0.012606 | 0.001027 | -0.62761 |
| PALTE6 | FC5 | O1 | 8 | 0 | 0.009617 | 0.001104 | -0.6246 |
| PALTEA6 | FC5 | O1 | 8 | 0 | 0.009617 | 0.001104 | -0.6246 |
| PALTA6 | FC5 | O1 | 16 | 1 | 0.012606 | 0.002016 | -0.59823 |
| DMSMLAD | F7 | AF4 | 128 | 2487.033 | 0.031164 | 0.002046 | 0.606957 |
| PALTA6 | FC5 | O1 | 8 | 1 | 0.009617 | 0.002591 | -0.58654 |
| DMSML4 | F7 | AF4 | 128 | 2475.667 | 0.031164 | 0.00267 | 0.593913 |
| DMSML | F7 | AF4 | 128 | 2820.375 | 0.031164 | 0.003748 | 0.576522 |
| DMSML4 | F7 | AF4 | 64 | 2475.667 | 0.020366 | 0.003937 | 0.573913 |
| PRMMDCLD | P7 | O1 | 128 | 1531 | 0.014275 | 0.004068 | -0.57217 |
| SWMTE12 | F3 | O2 | 32 | 14 | 0.012647 | 0.004842 | 0.555387 |
| PRMMCLD | P7 | O1 | 128 | 1667.148 | 0.014275 | 0.005592 | -0.55478 |
| PALTEA12 | O2 | T8 | 8 | 5.5 | 0.009039 | 0.00562 | 0.547495 |
| PALTE6 | F3 | F8 | 16 | 0 | 0.014418 | 0.005946 | -0.54446 |
| PALTEA6 | F3 | F8 | 16 | 0 | 0.014418 | 0.005946 | -0.54446 |
| PALTA6 | O1 | P8 | 8 | 1 | 0.011888 | 0.006302 | -0.54131 |
| PALTEA12 | F3 | P8 | 8 | 5.5 | 0.006001 | 0.006763 | 0.53743 |
| PALTA6 | F3 | F8 | 16 | 1 | 0.014418 | 0.006848 | -0.53673 |
| RVPAPercentile | AF3 | F3 | 64 | 59.95833 | 0.015412 | 0.007009 | -0.53545 |
| RVPAZScore | AF3 | F3 | 64 | 0.345417 | 0.015412 | 0.007009 | -0.53545 |
| DMSML12 | F7 | AF4 | 128 | 2846.1 | 0.031164 | 0.00757 | 0.537391 |
| PALTE6 | F3 | T7 | 16 | 0 | 0.012727 | 0.007734 | -0.52993 |
| PALTEA6 | F3 | T7 | 16 | 0 | 0.012727 | 0.007734 | -0.52993 |
| DMSMDL | F7 | AF4 | 128 | 2284 | 0.031164 | 0.007797 | 0.535652 |
| DMSMDLAD | F7 | AF4 | 128 | 2287.75 | 0.031164 | 0.008147 | 0.533043 |
| DMSML12 | F3 | O2 | 16 | 2846.1 | 0.009235 | 0.008267 | 0.532174 |
| DMSMLAD | F7 | AF4 | 64 | 2487.033 | 0.020366 | 0.008267 | 0.532174 |
| PRMMCLD | AF3 | O2 | 64 | 1667.148 | 0.010702 | 0.008635 | -0.52957 |
| SWMBE12 | F3 | O2 | 32 | 14 | 0.012647 | 0.009055 | 0.520907 |
| SWMSX | F7 | AF4 | 32 | 11.375 | 0.016554 | 0.009608 | -0.51746 |
| PALTA6 | F3 | T7 | 16 | 1 | 0.012727 | 0.009614 | -0.51742 |
| PRMMDCLD | P7 | O1 | 64 | 1531 | 0.009634 | 0.010105 | -0.52 |
| DMSML | F7 | AF4 | 64 | 2820.375 | 0.020366 | 0.010393 | 0.518261 |
| PALTE6 | O1 | P8 | 8 | 0 | 0.011888 | 0.010823 | -0.5104 |
| PALTEA6 | O1 | P8 | 8 | 0 | 0.011888 | 0.010823 | -0.5104 |
| RTIFRTSD | P7 | O1 | 128 | 36.54223 | 0.014275 | 0.011778 | -0.51043 |
| PRMMCLD | P7 | O1 | 64 | 1667.148 | 0.009634 | 0.011941 | -0.50957 |
| DMSML12 | AF3 | F4 | 16 | 2846.1 | 0.010502 | 0.012105 | 0.508696 |
| DMSMDL12 | F7 | AF4 | 128 | 2513.25 | 0.031164 | 0.012783 | 0.505217 |
| DMSL4SD | F7 | AF4 | 128 | 827.6409 | 0.031164 | 0.012958 | 0.504348 |
| PALTE6 | T8 | FC6 | 16 | 0 | 0.011573 | 0.013729 | -0.49587 |
| PALTEA6 | T8 | FC6 | 16 | 0 | 0.011573 | 0.013729 | -0.49587 |
| DMSL4SD | AF3 | F4 | 16 | 827.6409 | 0.010502 | 0.013859 | 0.5 |
| RVPPH | AF3 | F3 | 64 | 0.743063 | 0.015412 | 0.014045 | -0.49445 |
| RVPTH | AF3 | F3 | 64 | 40.125 | 0.015412 | 0.014045 | -0.49445 |
| RVPTM | AF3 | F3 | 64 | 13.875 | 0.015412 | 0.014045 | 0.494451 |
| PALTA6 | T8 | FC6 | 16 | 1 | 0.011573 | 0.014369 | -0.49302 |
| RVPMDL | FC6 | F4 | 128 | 411.5 | 0.025091 | 0.015009 | -0.49478 |
| RTIFMRT | FC6 | F4 | 128 | 341.5054 | 0.025091 | 0.015208 | -0.49391 |
| DMSMDL | F7 | AF4 | 64 | 2284 | 0.020366 | 0.01541 | 0.493043 |
| SWMS | T7 | FC6 | 8 | 6.708333 | 0.007192 | 0.015864 | 0.486738 |
| SWMSX | T7 | FC6 | 8 | 11.375 | 0.007192 | 0.016147 | 0.485605 |
| RVPA | AF3 | F3 | 64 | 0.9398 | 0.015412 | 0.017434 | -0.48064 |
| RTIFMDRT | FC6 | F4 | 128 | 334.3542 | 0.025091 | 0.017966 | -0.47868 |
| DMSMDLAD | F7 | AF4 | 64 | 2287.75 | 0.020366 | 0.018704 | 0.48 |
| RVPMDL | P7 | O1 | 64 | 411.5 | 0.009634 | 0.020174 | -0.47478 |
| RTIFMRT | AF3 | F3 | 64 | 341.5054 | 0.015412 | 0.020174 | 0.474783 |
| RTIFMDRT | AF3 | F3 | 64 | 334.3542 | 0.015412 | 0.020485 | 0.469974 |
| DMSMDL12 | AF3 | F4 | 16 | 2513.25 | 0.010502 | 0.021206 | 0.471304 |
| DMSML4 | O1 | FC6 | 128 | 2475.667 | 0.020816 | 0.021206 | 0.471304 |
| SWMBE12 | F3 | F8 | 16 | 14 | 0.014418 | 0.02144 | -0.4669 |
| RTIFRTSD | F7 | AF4 | 128 | 36.54223 | 0.031164 | 0.022008 | 0.468696 |
| PALTE8 | T7 | T8 | 8 | 4.041667 | 0.010855 | 0.02228 | 0.464286 |
| PALTEA8 | T7 | T8 | 8 | 4.041667 | 0.010855 | 0.02228 | 0.464286 |
| SWMS | F7 | AF4 | 32 | 6.708333 | 0.016554 | 0.022844 | -0.46258 |
| SWMS6 | F7 | AF4 | 32 | 3 | 0.016554 | 0.023409 | -0.46089 |
| DMSL4SD | F7 | AF4 | 64 | 827.6409 | 0.020366 | 0.023976 | 0.462609 |
| RTIFRTSD | P7 | O1 | 64 | 36.54223 | 0.009634 | 0.024564 | -0.46087 |
| SWMTE468 | F3 | F8 | 32 | 6.375 | 0.017748 | 0.025891 | 0.453884 |
| RVPAPercentile | AF3 | O2 | 64 | 59.95833 | 0.010702 | 0.027446 | -0.44976 |
| RVPAZScore | AF3 | O2 | 64 | 0.345417 | 0.010702 | 0.027446 | -0.44976 |
| RTIFRTSD | T7 | FC6 | 8 | 36.54223 | 0.007192 | 0.028013 | 0.451304 |
| DMSML12 | F7 | AF4 | 64 | 2846.1 | 0.020366 | 0.028345 | 0.450435 |
| DMSMDL12 | F3 | O2 | 16 | 2513.25 | 0.009235 | 0.02868 | 0.449565 |
| PALTE6 | F3 | F4 | 8 | 0 | 0.006184 | 0.029833 | -0.44378 |
| PALTEA6 | F3 | F4 | 8 | 0 | 0.006184 | 0.029833 | -0.44378 |
| SWMBE468 | F3 | F8 | 32 | 5 | 0.017748 | 0.029889 | 0.443648 |
| RVPAPercentile | AF3 | F3 | 8 | 59.95833 | 0.005407 | 0.030789 | -0.4415 |
| RVPAZScore | AF3 | F3 | 8 | 0.345417 | 0.005407 | 0.030789 | -0.4415 |
| PALTE8 | FC6 | F8 | 8 | 4.041667 | 0.007246 | 0.031353 | 0.440173 |
| PALTEA8 | FC6 | F8 | 8 | 4.041667 | 0.007246 | 0.031353 | 0.440173 |
| RVPMDL | P7 | O1 | 128 | 411.5 | 0.014275 | 0.032584 | -0.44 |
| SWMTE12 | F3 | F8 | 16 | 14 | 0.014418 | 0.033522 | -0.43526 |
| PRMMCLI | P7 | O1 | 128 | 1516.364 | 0.014275 | 0.03372 | -0.43739 |
| PALTEA12 | AF3 | F3 | 8 | 5.5 | 0.005407 | 0.036082 | -0.42977 |
| RVPML | O1 | P8 | 128 | 466.6207 | 0.027079 | 0.037321 | -0.42957 |
| PRMMCLI | AF3 | O2 | 64 | 1516.364 | 0.010702 | 0.037321 | -0.42957 |
| SWMBE12 | FC5 | AF4 | 8 | 14 | 0.010111 | 0.037731 | -0.42639 |
| SWMTE12 | FC5 | AF4 | 8 | 14 | 0.010111 | 0.038526 | -0.42481 |
| RTIFMRT | F7 | AF4 | 128 | 341.5054 | 0.031164 | 0.038588 | 0.426957 |
| DMSL4SD | F3 | O2 | 32 | 827.6409 | 0.012647 | 0.038588 | 0.426957 |
| DMSMDLS | T7 | FC6 | 8 | 2252.5 | 0.007192 | 0.03983 | -0.42227 |
| SWMS6 | FC6 | F4 | 128 | 3 | 0.025091 | 0.040598 | -0.4208 |
| SWMBE468 | T7 | FC6 | 8 | 5 | 0.007192 | 0.040713 | 0.420578 |
| SWMTE468 | T7 | FC6 | 8 | 6.375 | 0.007192 | 0.040915 | 0.420198 |
| PALTE28 | O2 | T8 | 8 | 5 | 0.009039 | 0.042519 | 0.417218 |
| PALTEA28 | O2 | T8 | 8 | 5 | 0.009039 | 0.042519 | 0.417218 |
| RVPMDL | O1 | P8 | 128 | 411.5 | 0.027079 | 0.043531 | -0.41739 |
| RTIFMDRT | F7 | AF4 | 128 | 334.3542 | 0.031164 | 0.043665 | 0.415144 |
| PALFAMS28 | F7 | AF4 | 32 | 16.54167 | 0.016554 | 0.043888 | 0.414745 |
| RVPMDL | T7 | AF4 | 8 | 411.5 | 0.009495 | 0.045938 | 0.413043 |
| PALTA28 | O2 | T8 | 8 | 6 | 0.009039 | 0.046821 | 0.409642 |
| PALTEA12 | O1 | P8 | 64 | 5.5 | 0.019597 | 0.047342 | 0.408762 |
| DMSML | F3 | O2 | 16 | 2820.375 | 0.009235 | 0.047939 | 0.409565 |
| DMSL4SD | T7 | AF4 | 8 | 827.6409 | 0.009495 | 0.047939 | -0.40957 |
| SWMTE468 | F7 | AF4 | 32 | 6.375 | 0.016554 | 0.048459 | -0.4069 |
| RTIFRTSD | F3 | F8 | 32 | 36.54223 | 0.017748 | 0.048966 | 0.407826 |
| DMSMDL | T7 | T8 | 64 | 2284 | 0.022262 | 0.049485 | 0.406957 |

**Supplementary Table S5.** All significant correlations between $\sigma_{DCCC\left( s \right)}^{2}$ and CANTAB scores in the elderly group.

| CANTAB | ch_1_ | ch_2_ | scale | E_cantab | E_dccc | *p*-value | *r* |
| --- | --- | --- | --- | --- | --- | --- | --- |
| PALTE6 | FC6 | F4 | 8 | 3 | 0.007659 | 0.000128 | -0.76694 |
| PALTEA6 | FC6 | F4 | 8 | 3 | 0.007659 | 0.000128 | -0.76694 |
| DMSML12 | F7 | AF4 | 32 | 4322 | 0.020419 | 0.000184 | -0.76842 |
| PALTE6 | FC6 | F4 | 16 | 3 | 0.01061 | 0.000189 | -0.75456 |
| PALTEA6 | FC6 | F4 | 16 | 3 | 0.01061 | 0.000189 | -0.75456 |
| DMSMDL12 | F7 | AF4 | 32 | 3860 | 0.020419 | 0.000338 | -0.74912 |
| SWMTE468 | FC6 | F4 | 64 | 15.78947 | 0.015289 | 0.001274 | -0.6828 |
| DMSMDLAD | FC5 | F8 | 64 | 3586 | 0.02129 | 0.001278 | -0.69649 |
| DMSML12 | FC5 | AF4 | 8 | 4322 | 0.011918 | 0.00138 | -0.69298 |
| PALTEA12 | T7 | AF4 | 8 | 12 | 0.00791 | 0.001424 | -0.67793 |
| SWMSX | FC6 | F4 | 64 | 15.52632 | 0.015289 | 0.001573 | -0.67348 |
| SWMBE468 | FC6 | F4 | 64 | 19 | 0.015289 | 0.001575 | -0.67342 |
| DMSML | FC5 | F8 | 64 | 3677.531 | 0.02129 | 0.001605 | -0.68596 |
| DMSMLAD | FC5 | F8 | 64 | 3956.667 | 0.02129 | 0.002144 | -0.67193 |
| PALTA6 | FC6 | F4 | 16 | 2 | 0.01061 | 0.002171 | -0.65854 |
| SWMSX | FC5 | F8 | 64 | 15.52632 | 0.02129 | 0.002907 | -0.64427 |
| DMSMLS | AF3 | F3 | 64 | 3276.616 | 0.014872 | 0.003666 | 0.64386 |
| RTIFRTSD | T7 | T8 | 8 | 46.89996 | 0.007624 | 0.003666 | -0.64386 |
| PALMETS28 | O1 | FC6 | 128 | 2 | 0.017407 | 0.004067 | 0.626964 |
| PALTA6 | FC6 | F4 | 8 | 2 | 0.007659 | 0.004362 | -0.62323 |
| DMSML | FC5 | AF4 | 8 | 3677.531 | 0.011918 | 0.004426 | -0.63333 |
| PALTEA12 | F3 | T7 | 16 | 12 | 0.01242 | 0.004911 | -0.61678 |
| PALTE6 | T7 | AF4 | 8 | 3 | 0.00791 | 0.005689 | -0.6086 |
| PALTEA6 | T7 | AF4 | 8 | 3 | 0.00791 | 0.005689 | -0.6086 |
| PALTA6 | P8 | T8 | 32 | 2 | 0.009337 | 0.006362 | -0.60223 |
| DMSMDL | FC5 | F8 | 64 | 3459 | 0.02129 | 0.006517 | -0.61053 |
| PALTE6 | P8 | T8 | 16 | 3 | 0.006923 | 0.007604 | -0.59179 |
| PALTEA6 | P8 | T8 | 16 | 3 | 0.006923 | 0.007604 | -0.59179 |
| PALTA6 | P8 | T8 | 16 | 2 | 0.006923 | 0.008113 | -0.58792 |
| SWMS | FC5 | F8 | 64 | 8.947368 | 0.02129 | 0.008262 | -0.58682 |
| SWMBE4 | FC6 | F4 | 64 | 2 | 0.015289 | 0.008329 | -0.58633 |
| SWMTE4 | FC6 | F4 | 64 | 2 | 0.015289 | 0.008329 | -0.58633 |
| PALMETS28 | FC6 | F4 | 64 | 2 | 0.015289 | 0.00855 | -0.58474 |
| DMSMDL | AF3 | F3 | 8 | 3459 | 0.005303 | 0.009105 | 0.589474 |
| DMSMLAD | FC5 | AF4 | 8 | 3956.667 | 0.011918 | 0.009354 | -0.58772 |
| SWMBE4 | T7 | T8 | 32 | 2 | 0.016302 | 0.010949 | 0.569334 |
| SWMTE4 | T7 | T8 | 32 | 2 | 0.016302 | 0.010949 | 0.569334 |
| DMSMDL12 | F7 | AF4 | 64 | 3860 | 0.024018 | 0.010966 | -0.57719 |
| SWMWE8 | AF3 | F3 | 64 | 0 | 0.014872 | 0.01097 | 0.569212 |
| SWMSX | F3 | F8 | 32 | 15.52632 | 0.018942 | 0.011152 | -0.56816 |
| DMSMDL | O2 | T8 | 8 | 3459 | 0.008669 | 0.012475 | -0.56842 |
| DMSMDL12 | F7 | AF4 | 128 | 3860 | 0.032135 | 0.013459 | -0.56316 |
| DMSML12 | FC5 | O1 | 8 | 4322 | 0.011277 | 0.0138 | -0.5614 |
| SWMSX | F3 | F8 | 16 | 15.52632 | 0.015649 | 0.014032 | -0.55312 |
| PALTA6 | FC5 | O1 | 16 | 2 | 0.014235 | 0.014141 | -0.5526 |
| DMSMDL | AF3 | F3 | 64 | 3459 | 0.014872 | 0.014149 | 0.559649 |
| DMSMLAD | O2 | T8 | 8 | 3956.667 | 0.008669 | 0.014149 | -0.55965 |
| DMSML12 | F7 | AF4 | 64 | 4322 | 0.024018 | 0.014504 | -0.55789 |
| PALTE6 | FC5 | O1 | 16 | 3 | 0.014235 | 0.014651 | -0.55022 |
| PALTE6 | P8 | T8 | 32 | 3 | 0.009337 | 0.014651 | -0.55022 |
| PALTEA6 | FC5 | O1 | 16 | 3 | 0.014235 | 0.014651 | -0.55022 |
| PALTEA6 | P8 | T8 | 32 | 3 | 0.009337 | 0.014651 | -0.55022 |
| DMSML12 | FC5 | F8 | 64 | 4322 | 0.02129 | 0.014867 | -0.55614 |
| DMSMDLAD | FC5 | AF4 | 8 | 3586 | 0.011918 | 0.015237 | -0.55439 |
| DMSMDL12 | FC5 | AF4 | 8 | 3860 | 0.011918 | 0.016 | -0.55088 |
| PALTEA12 | FC6 | F4 | 16 | 12 | 0.01061 | 0.016653 | -0.54145 |
| DMSMDL12 | T7 | FC6 | 8 | 3860 | 0.006578 | 0.016793 | 0.547368 |
| DMSMDLS | AF3 | F3 | 64 | 3238.868 | 0.014872 | 0.018041 | 0.542105 |
| DMSL4SD | O1 | FC6 | 32 | 1456.715 | 0.010016 | 0.018041 | 0.542105 |
| RTIFMDRT | T7 | FC6 | 8 | 383.7895 | 0.006578 | 0.018118 | -0.53556 |
| DMSML | O2 | T8 | 8 | 3677.531 | 0.008669 | 0.018474 | -0.54035 |
| SWMWE8 | O2 | T8 | 8 | 0 | 0.008669 | 0.019175 | -0.53154 |
| PALTA28 | T7 | AF4 | 8 | 8 | 0.00791 | 0.019294 | -0.53109 |
| DMSMDL | FC5 | AF4 | 8 | 3459 | 0.011918 | 0.019363 | -0.53684 |
| PALTE4 | F3 | O2 | 32 | 0 | 0.012042 | 0.020073 | 0.528252 |
| PALTEA4 | F3 | O2 | 32 | 0 | 0.012042 | 0.020073 | 0.528252 |
| SWMWE8 | FC6 | F4 | 8 | 0 | 0.007659 | 0.020294 | -0.52746 |
| PALTE4 | T7 | T8 | 32 | 0 | 0.016302 | 0.020646 | 0.526221 |
| PALTEA4 | T7 | T8 | 32 | 0 | 0.016302 | 0.020646 | 0.526221 |
| PALFAMS28 | FC6 | F4 | 64 | 11.94737 | 0.015289 | 0.0234 | 0.517027 |
| SWMSX | FC6 | F4 | 8 | 15.52632 | 0.007659 | 0.023461 | -0.51683 |
| PRMMCLI | O1 | FC6 | 16 | 1900.687 | 0.007303 | 0.023804 | -0.52105 |
| RTIFRTSD | FC5 | F8 | 64 | 46.89996 | 0.02129 | 0.023804 | 0.521053 |
| PRMMCLD | O1 | FC6 | 32 | 2082.149 | 0.010016 | 0.024344 | -0.5193 |
| PALTA28 | FC6 | F4 | 16 | 8 | 0.01061 | 0.024615 | -0.51324 |
| PALTA4 | F3 | F4 | 8 | 1 | 0.007058 | 0.025033 | -0.51197 |
| PALTE4 | F3 | F4 | 8 | 0 | 0.007058 | 0.025363 | -0.51098 |
| PALTEA4 | F3 | F4 | 8 | 0 | 0.007058 | 0.025363 | -0.51098 |
| RVPA | T7 | T8 | 16 | 0.8971 | 0.01046 | 0.026948 | 0.506362 |
| PALTA6 | T7 | AF4 | 8 | 2 | 0.00791 | 0.027134 | -0.50584 |
| RTIFMRT | T7 | FC6 | 8 | 391.4468 | 0.006578 | 0.027192 | -0.51053 |
| PRMMCLD | T7 | T8 | 8 | 2082.149 | 0.007624 | 0.027192 | -0.51053 |
| DMSML4 | FC5 | F8 | 64 | 3634.2 | 0.02129 | 0.027792 | -0.50877 |
| PALFAMS28Percentile | FC6 | F4 | 64 | 44.05263 | 0.015289 | 0.028044 | 0.503294 |
| PALFAMS28ZScore | FC6 | F4 | 64 | -0.16842 | 0.015289 | 0.028044 | 0.503294 |
| PALTA28 | T7 | T8 | 32 | 8 | 0.016302 | 0.028322 | 0.50253 |
| DMSMDL12 | FC5 | O1 | 8 | 3860 | 0.011277 | 0.029024 | -0.50526 |
| PALFAMS28Percentile | P8 | FC6 | 64 | 44.05263 | 0.020001 | 0.02934 | 0.499781 |
| PALFAMS28ZScore | P8 | FC6 | 64 | -0.16842 | 0.020001 | 0.02934 | 0.499781 |
| PALTA4 | T7 | T8 | 32 | 1 | 0.016302 | 0.029367 | 0.499709 |
| PALTA4 | F3 | O2 | 32 | 1 | 0.012042 | 0.030537 | 0.496643 |
| PALTEA12 | FC6 | F4 | 8 | 12 | 0.007659 | 0.030686 | -0.49626 |
| DMSMDL12 | AF3 | F3 | 8 | 3860 | 0.005303 | 0.032294 | 0.496491 |
| DMSMLAD | F7 | O2 | 16 | 3956.667 | 0.008226 | 0.032294 | -0.49649 |
| SWMTE468 | FC6 | F4 | 128 | 15.78947 | 0.021762 | 0.033519 | -0.48922 |
| DMSMLAD | F7 | AF4 | 32 | 3956.667 | 0.020419 | 0.034392 | -0.49123 |
| DMSMDLAD | O2 | T8 | 8 | 3586 | 0.008669 | 0.034392 | -0.49123 |
| SWMS | FC6 | F4 | 64 | 8.947368 | 0.015289 | 0.035436 | -0.48472 |
| DMSML12 | FC5 | O1 | 16 | 4322 | 0.014235 | 0.035849 | -0.48772 |
| DMSL4SD | F7 | O2 | 16 | 1456.715 | 0.008226 | 0.037354 | -0.48421 |
| SWMBE468 | FC6 | F4 | 128 | 19 | 0.021762 | 0.03805 | -0.47888 |
| SWMSX | FC6 | F4 | 128 | 15.52632 | 0.021762 | 0.038094 | -0.47878 |
| PALMETS28 | FC6 | F4 | 128 | 2 | 0.021762 | 0.038762 | -0.47734 |
| RVPPH | T7 | T8 | 16 | 0.614037 | 0.01046 | 0.038866 | 0.477116 |
| RVPTH | T7 | T8 | 16 | 33.15789 | 0.01046 | 0.038866 | 0.477116 |
| RVPTM | T7 | T8 | 16 | 20.84211 | 0.01046 | 0.038866 | -0.47712 |
| SWMBE6 | AF3 | O2 | 64 | 6 | 0.010431 | 0.039377 | 0.476027 |
| PALTEA12 | T7 | T8 | 64 | 12 | 0.022105 | 0.040292 | 0.474105 |
| SWMWE8 | FC6 | F4 | 16 | 0 | 0.01061 | 0.042082 | -0.47044 |
| PALTEA12 | O2 | T8 | 8 | 12 | 0.008669 | 0.042463 | -0.46967 |
| PALTA4 | O1 | FC6 | 32 | 1 | 0.010016 | 0.042775 | -0.46905 |
| SWMBE12 | FC6 | F4 | 64 | 35 | 0.015289 | 0.043225 | -0.46816 |
| DMSMLS | F3 | T7 | 128 | 3276.616 | 0.025618 | 0.044756 | 0.468421 |
| SWMS6 | T7 | T8 | 64 | 4 | 0.022105 | 0.046038 | 0.462745 |
| SWMS | F3 | F8 | 16 | 8.947368 | 0.015649 | 0.046154 | -0.46253 |
| PALTA4 | T8 | FC6 | 8 | 1 | 0.006956 | 0.04649 | -0.4619 |
| DMSMDLS | T7 | T8 | 64 | 3238.868 | 0.022105 | 0.046546 | 0.464912 |
| DMSMLS | AF3 | O2 | 64 | 3276.616 | 0.010431 | 0.046546 | 0.464912 |
| PRMMDCLD | O1 | FC6 | 32 | 1927.842 | 0.010016 | 0.046546 | -0.46491 |
| SWMBE4 | AF3 | O2 | 64 | 2 | 0.010431 | 0.046597 | 0.461699 |
| SWMTE4 | AF3 | O2 | 64 | 2 | 0.010431 | 0.046597 | 0.461699 |
| SWMTE12 | O1 | FC6 | 128 | 35 | 0.017407 | 0.046647 | 0.461606 |
| PALTA28 | FC6 | F4 | 64 | 8 | 0.015289 | 0.047202 | -0.46058 |
| SWMBE12 | AF3 | O2 | 128 | 35 | 0.014886 | 0.047857 | 0.459377 |
| PRMMDCLI | AF3 | O2 | 64 | 1744 | 0.010431 | 0.048078 | 0.458973 |
| PRMMDCLD | T8 | FC6 | 8 | 1927.842 | 0.006956 | 0.048392 | -0.4614 |
| SWMSX | FC5 | AF4 | 8 | 15.52632 | 0.011918 | 0.048871 | -0.45754 |
| PALTA6 | T8 | FC6 | 64 | 2 | 0.016094 | 0.049082 | 0.457162 |
| DMSMDLS | FC6 | F4 | 64 | 3238.868 | 0.015289 | 0.049336 | -0.45965 |
| DMSMLS | AF3 | F3 | 8 | 3276.616 | 0.005303 | 0.049336 | 0.459649 |

**Supplementary Table S6.** All significant correlations between $\mu_{DFA}$ and CANTAB scores in the young group.

| CANTAB | ch | E_cantab | E_DFA | *p*-value | *r* |
| --- | --- | --- | --- | --- | --- |
| DMSL4SD | AF4 | 827.6409 | 0.889141 | 0.01645 | 0.488696 |
| DMSL4SD | P7 | 827.6409 | 0.8376 | 0.029018 | 0.448696 |
| DMSML4 | AF4 | 2475.667 | 0.889141 | 0.036907 | 0.430435 |
| RVPA | F4 | 0.9398 | 0.885202 | 0.042089 | -0.41801 |
| RVPAPercentile | F4 | 59.95833 | 0.885202 | 0.042563 | -0.41714 |
| RVPAZScore | F4 | 0.345417 | 0.885202 | 0.042563 | -0.41714 |
| RVPPH | F4 | 0.743063 | 0.885202 | 0.043372 | -0.41567 |
| RVPTH | F4 | 40.125 | 0.885202 | 0.043372 | -0.41567 |
| RVPTM | F4 | 13.875 | 0.885202 | 0.043372 | 0.415669 |

**Supplementary Table S7.** All significant correlations between $\mu_{DFA}$ and CANTAB scores in the elderly group.

| CANTAB | ch | E_cantab | E_DFA | *p*-value | r |
| --- | --- | --- | --- | --- | --- |
| RVPAPercentile | P8 | 59.95833 | 0.779142 | 0.000817 | -0.70149 |
| RVPAZScore | P8 | 0.345417 | 0.779142 | 0.000817 | -0.70149 |
| RVPA | P8 | 0.9398 | 0.779142 | 0.001814 | -0.66696 |
| RVPA | AF4 | 0.9398 | 0.806229 | 0.004743 | -0.61869 |
| RVPPH | P8 | 0.743063 | 0.779142 | 0.005902 | -0.60652 |
| RVPTH | P8 | 40.125 | 0.779142 | 0.005902 | -0.60652 |
| RVPTM | P8 | 13.875 | 0.779142 | 0.005902 | 0.606518 |
| PALFAMS28Percentile | F4 | 66.5 | 0.821163 | 0.007803 | 0.590251 |
| PALFAMS28ZScore | F4 | 0.624167 | 0.821163 | 0.007803 | 0.590251 |
| RVPAPercentile | AF4 | 59.95833 | 0.806229 | 0.008429 | -0.5856 |
| RVPAZScore | AF4 | 0.345417 | 0.806229 | 0.008429 | -0.5856 |
| RTIFRTSD | AF4 | 36.54223 | 0.806229 | 0.008862 | 0.591228 |
| RVPA | O1 | 0.9398 | 0.772706 | 0.012334 | -0.56165 |
| RVPPH | AF4 | 0.743063 | 0.806229 | 0.016046 | -0.54402 |
| RVPTH | AF4 | 40.125 | 0.806229 | 0.016046 | -0.54402 |
| RVPTM | AF4 | 13.875 | 0.806229 | 0.016046 | 0.544017 |
| PALFAMS28 | F4 | 16.54167 | 0.821163 | 0.018339 | 0.534704 |
| RTIFMDRT | O1 | 334.3542 | 0.772706 | 0.021501 | -0.52327 |
| RVPPH | O1 | 0.743063 | 0.772706 | 0.021612 | -0.52289 |
| RVPTH | O1 | 40.125 | 0.772706 | 0.021612 | -0.52289 |
| RVPTM | O1 | 13.875 | 0.772706 | 0.021612 | 0.522891 |
| RVPAPercentile | O1 | 59.95833 | 0.772706 | 0.025365 | -0.51097 |
| RVPAZScore | O1 | 0.345417 | 0.772706 | 0.025365 | -0.51097 |
| RTIFMDRT | P7 | 334.3542 | 0.782462 | 0.02718 | -0.50571 |
| PALFAMS28Percentile | T7 | 66.5 | 0.788368 | 0.034624 | 0.486606 |
| PALFAMS28ZScore | T7 | 0.624167 | 0.788368 | 0.034624 | 0.486606 |
| PRMMCLD | T8 | 1667.148 | 0.770546 | 0.035849 | -0.48772 |
| RTIFRTSD | O1 | 36.54223 | 0.772706 | 0.044756 | 0.468421 |
| PALFAMS28Percentile | AF4 | 66.5 | 0.806229 | 0.045496 | 0.463769 |
| PALFAMS28ZScore | AF4 | 0.624167 | 0.806229 | 0.045496 | 0.463769 |
| PRMMDCLD | T8 | 1531 | 0.770546 | 0.045644 | -0.46667 |
| RTIFRTSD | F4 | 36.54223 | 0.821163 | 0.046546 | 0.464912 |
| RVPAPercentile | FC6 | 59.95833 | 0.788448 | 0.047487 | -0.46005 |
| RVPAZScore | FC6 | 0.345417 | 0.788448 | 0.047487 | -0.46005 |
| RTIFMRT | P7 | 341.5054 | 0.782462 | 0.049336 | -0.45965 |
